# Gas41 links histone acetylation to H2A.Z deposition and maintenance of embryonic stem cell identity

**DOI:** 10.1038/s41421-018-0027-0

**Published:** 2018-06-12

**Authors:** Chih-Chao Hsu, Dan Zhao, Jiejun Shi, Danni Peng, Haipeng Guan, Yuanyuan Li, Yaling Huang, Hong Wen, Wei Li, Haitao Li, Xiaobing Shi

**Affiliations:** 1 Department of Epigenetics and Molecular Carcinogenesis and Center for Cancer Epigenetics, The University of Texas MD Anderson Cancer Center, Houston, TX 77030 USA; 2 MOE Key Laboratory of Protein Sciences, Beijing Advanced Innovation Center for Structural Biology, Department of Basic Medical Sciences, School of Medicine, Tsinghua University, Beijing, 100084 China; 3 Tsinghua-Peking Joint Center for Life Sciences, Tsinghua University, Beijing, 100084 China; 4 Dan L. Duncan Cancer Center, Department of Molecular and Cellular Biology, Baylor College of Medicine, Houston, TX 77030 USA; 5 Genetics and Epigenetics Graduate Program, The University of Texas MD Anderson Cancer Center UTHealth Graduate School of Biomedical Sciences, Houston, TX 77030 USA

## Abstract

The histone variant H2A.Z is essential for maintaining embryonic stem cell (ESC) identity in part by keeping developmental genes in a poised bivalent state. However, how H2A.Z is deposited into the bivalent domains remains unknown. In mammals, two chromatin remodeling complexes, Tip60/p400 and SRCAP, exchange the canonical histone H2A for H2A.Z in the chromatin. Here we show that Glioma Amplified Sequence 41 (Gas41), a shared subunit of the two H2A.Z-depositing complexes, functions as a reader of histone lysine acetylation and recruits Tip60/p400 and SRCAP to deposit H2A.Z into specific chromatin regions including bivalent domains. The YEATS domain of Gas41 bound to acetylated histone H3K27 and H3K14 both in vitro and in cells. The crystal structure of the Gas41 YEATS domain in complex with the H3K27ac peptide revealed that, similar to the AF9 and ENL YEATS domains, Gas41 YEATS forms a serine-lined aromatic cage for acetyllysine recognition. Consistently, mutations in the aromatic residues of the Gas41 YEATS domain abrogated the interaction. In mouse ESCs, knockdown of Gas41 led to flattened morphology of ESC colonies, as the result of derepression of differentiation genes. Importantly, the abnormal morphology was rescued by expressing wild-type Gas41, but not the YEATS domain mutated counterpart that does not recognize histone acetylation. Mechanically, we found that Gas41 depletion led to reduction of H2A.Z levels and a concomitant reduction of H3K27me3 levels on bivalent domains. Together, our study reveals an essential role of the Gas41 YEATS domain in linking histone acetylation to H2A.Z deposition and maintenance of ESC identity.

## Introduction

Embryonic stem cells (ESCs) are characterized by two features: self-renewal, the ability to propagate themselves indefinitely; and pluripotency, the ability to differentiate into all cell types of the body^1^. The self-renewal feature is maintained by the transcriptional circuitry of core pluripotent factors, such as Oct4, Sox2 and Nanog. However, during differentiation, the expression of these core pluripotent factors is abrogated and replaced by lineage-specific transcription factors^2^. A growing body of evidence suggests that in addition to transcription factors, epigenetic mechanisms, such as posttranslational modifications (PTMs) of histones and histone variants, also play a critical role in these processes^2–4^. For instance, in ESCs, promoters of many developmental genes are characterized by the coexistence of two functionally opposite epigenetic marks, the active H3 lysine 4 trimethylation (H3K4me3) mark and the repressive H3 K27 trimethylation (H3K27me3) mark, thus creating a “bivalent domain”^5,6^. Such domains allow both the suppression of developmental genes during the undifferentiated stage, and the rapid induction of lineage specification genes in response to differentiation cues^7^.

Histone variants are also important players in ESC self-renewal and differentiation, such as H2A.Z, an evolutionarily conserved histone H2A variant. In vertebrates, there are two H2A.Z isoforms, H2AZ.1 and H2AZ.2, which differ from each other by only three amino acids^8,9^. H2A.Z.1 is essential for early mammalian development; knockout of H2A.Z.1 causes embryonic lethality around the time of implantation^10^. Consistent with an important developmental role, H2A.Z is required for the maintenance of mouse ESC (mESC) identity^11–13^, in part through its role in regulating gene expression at bivalent domains. The bivalent promoter-associated H2A.Z is required for efficient recruitment of the Polycomb repressive complex 2 (PRC2), which deposits the repressive H3K27me3 mark. As a result, knockdown (KD) of H2A.Z leads to derepression of bivalent genes^11,14^.

In contrast to canonical histone H2A, which is distributed across the genome and lacks a specific localization pattern, H2A.Z is enriched in regulatory regions such as promoters and enhancers^11,15,16^. Deposition of H2A.Z into chromatin is catalyzed by two variant specific complexes: the SRCAP (Snf2-related CBP activator protein) and Tip60/p400 complexes^17–19^. The two complexes share a number of subunits, including Gas41 (Glioma-amplified sequence 41), a YEATS domain-containing protein. Recently, we identified the YEATS domains of human AF9 and ENL proteins as novel readers of histone acetylation^20,21^. However, the role of the Gas41 YEATS domain remains unknown. Here we show that the Gas41 YEATS domain is a histone acetylation reader. Distinct from the YEATS domains of AF9 and ENL, which specifically recognize acetylation of lysine in the “RK” motif, the Gas41 YEATS domain is able to bind to acetylation on both H3K27 and H3K14. Importantly, the acetylation reader function of the YEATS domain is important for Gas41 function in mESCs. Disruption of the acetylation recognition activity of the Gas41 YEATS domain leads to a reduction in chromatin associated H2A.Z and a concomitant reduction of H3K27me3 in the bivalent domains, and consequently, derepression of developmental genes. Collectively, our results uncover a critical role for the Gas41 YEATS domain in the maintenance of ESC identity by linking histone acetylation to H2A.Z deposition.

## Results

### Gas41 is required for maintenance of mESC identity

**Fig. 1:**
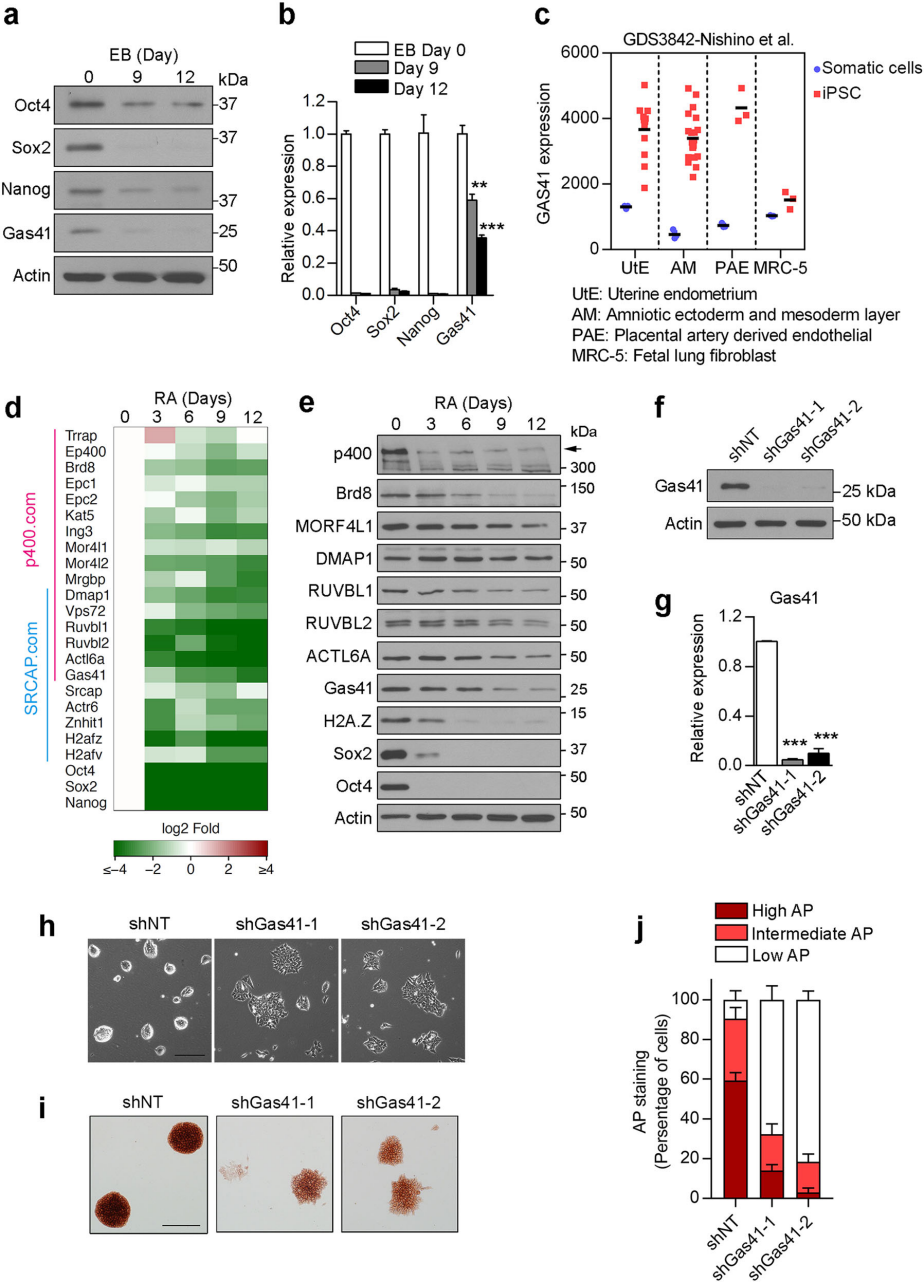
Gas41 is highly expressed in undifferentiated mESCs. **a** Western blot analysis of Gas41 and the pluripotent factors Oct4, Sox2 and Nanog at the specified times during embryoid body (EB) formation. β-Actin serves as a loading control. **b** RT–PCR analysis of *Gas41, Oct4, Sox2* and *Nanog* expression levels during EB formation. Data was normalized to *Actb* with EB day 0 set as 1. Error bars represent the SEM from n = 3 biologically independent experiments. **P < 0.05; ***P < 0.001. Two-tailed unpaired Student’s *t*-test. **c** Dotplot depicting Gas41 mRNA levels in induced pluripotent stem cells (iPSC) and their parental somatic cells. Data were extracted from the Gene Expression Omnibus database GDS3842^49^. **d** Heatmap showing change in gene expression (determined by qRT–PCR performed in technical duplicate) of Tip60/p400 and SRCAP complexes components on indicate day of RA treatment. Shown are log2FC gene expression at indicated day of treatment compared to day 0 (undifferentiated mESC). **e** Western blot analysis protein expression of indicated Tip60/p400 and SRCAP complexes components in RA treated mESCs. Arrow head, p400. β-Actin serves as a loading control. **f** Western blot analysis of Gas41 in control (shNT) and Gas41 KD (shGas41) mouse J1 ESCs. **g** RT–PCR analysis of *Gas41* gene expression in control and Gas41 KD mESCs. Error bars represent the SEM from n = 3 biologically independent experiments. ***P < 0.001 (compared with shNT, Two-tailed Student’s *t*-test). **h** Phase contrast images of control and Gas41 KD mESCs. Bar, 200 μm. **i** Alkaline phosphate (AP) staining of control and Gas41 KD J1 cells. Bar, 200 µm. **j** Stacked bar plot showing quantification of AP staining from **i**. Error bars represent the SEM from 6 (shGas41-1) or 7 (shNT and shGas41-2) randomly selected fields. Statistical analyses are available in Supplementary Table S7

Core pluripotent transcriptional factors, such as Oct4, Sox2 and Nanog, are essential for mESC self-renewal but are turned off during differentiation to allow lineage-specific developmental gene expression^22–26^. During the course of embryoid body (EB) differentiation assays using J1 mouse ESCs, we observed a rapid deactivation of Oct4, Sox2 and Nanog (Fig. 1a, b) and a decrease in both Gas41 protein and mRNA levels, although less dramatic than that of the core pluripotent factors. Forced mESC differentiation by short hairpin RNA (shRNA)-mediated depletion of Oct4 also led to downregulation of Gas41 along with Sox2 and Nanog (Supplementary Figure S1a, b). Similar to the core pluripotent transcriptional factors, Gas41 was expressed at a much lower level in mouse embryonic fibroblasts (MEFs) than in mESCs (Supplementary Figure S1c, d). Furthermore, analyses of several Gene Expression Omnibus (GEO) datasets also revealed that Gas41 gene expression was downregulated during EB formation or lineage-specific differentiation (Supplementary Figure S1e–h); whereas markedly upregulated when somatic cells were reprogrammed to the induced pluripotent stem cells (iPSCs) (Fig. 1c and Supplementary Figure S1i-k). Gas41 is a stoichiometric component of the Tip60/p400 and SRCAP complexes^17–19^. We thus also determined the expression of genes encoding these two complexes during differentiation, and we found that almost all the genes were downregulated during retinoid acid (RA)-induced differentiation of J1 mESCs (Fig. 1d, e).

These data indicate that Gas41 is required for maintenance of ESC pluripotency. To test this hypothesis, we knocked down Gas41 by two independent shRNAs (Fig. 1f, g) and assessed ESC differentiation status. We found that Gas41 KD cells exhibited a flattened morphology (Fig. 1h) and reduced alkaline phosphatase (AP) activity (Fig. 1i, j). We observed similar phenotypes by CRISPR-Cas9-mediated knockout (KO) of Gas41 in J1 mESCs (Supplementary Figure S1l-n), Together, these data suggest that Gas41 is highly expressed in undifferentiated mESCs and is required for maintenance of mESC identity.

### Gas41 suppresses developmental genes in mESCs

**Fig. 2:**
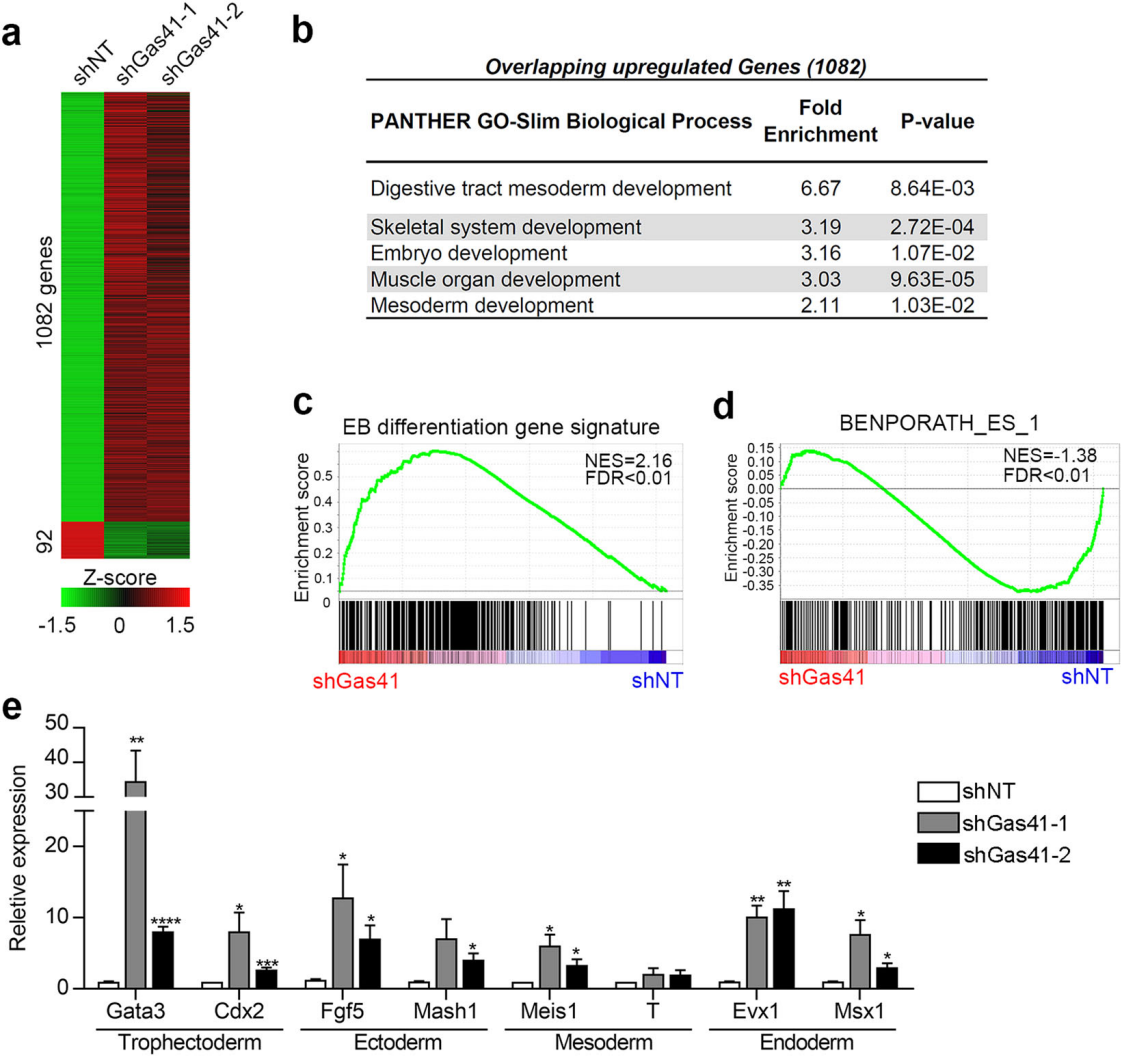
Gas41 knockdown induces differentiation gene expression in mESCs. **a** Heatmap representation of differentially expressed genes [absolute log2FC (fold change) ≥ 1 and FDR ≤ 0.05] in control (shNT) and Gas41 KD (shGas41-1 and shGas41-2) mESCs. **b** GO analysis of overlapping upregulated genes in shGas41-1 and shGas41-2 treated mESCs. The top five enriched process are listed. The full lists of are provided in Supplementary Table S2. **c** Gene Set Enrichment Analysis (GSEA) plot of EB differentiation gene signature (for definition, see Supplementary Figure S2a) in shGas41 treated mESCs. FDR, false discovery rate; NES, normalized enrichment score. **d** GSEA plot of ESC gene signature (Data set: BENPORATH_ES_1, which includes 379 genes overexpressed in human embryonic stem cells) in shGas41 treated mESCs. **e** RT–PCR analysis of expression of selected lineage marker genes in control (shNT) and Gas41 KD mESCs. Error bars represent the SEM from *n* = 4 biologically independent experiments. *P < 0.05, **P < 0.01, ***P < 0.001, ****P < 0.0001 (compared with shNT, Two-tailed Student’s *t*-test). Statistics source data are shown in Supplementary Table S7

To gain insights into global gene expression regulated by Gas41, we performed RNA sequencing (RNA-seq) experiments in Gas41 KD (shGas41-1 and shGas41-2) mESCs compared to mock KD (shNT) mESCs. We identified total 1082 genes upregulated and 92 genes downregulated (fold change ≥2, FDR ≤ 0.05) in both KD cells (Fig. 2a, Supplementary Figure S2a and Supplementary Table S1). The greater number of upregulated genes vs. downregulated genes suggests that Gas41 preferentially functions as a transcriptional co-repressor in mESCs. Gene ontology analysis with PANTHER^27,28^ revealed that the 1082 upregulated genes were enriched in developmental processes (Fig. 2b and Supplementary Table S1), whereas, the 92 downregulated genes were not enriched in any biological process. We also performed Gene Set Enrichment Analysis (GSEA) of gene expression profiles in Gas41 KD ESCs to evaluate their enrichment in differentiation vs ESC signature genes. Using an “EB differentiation gene signature” defined by 499 genes highly upregulated (Log_2_FC ≥ 5) in day 6 EBs compared to the undifferentiated mESCs (Supplementary Figure S2b and Supplementary Table S2), we found a strong enrichment of EB differentiation genes in Gas41 KD cells vs. control cells (Fig. 2c), whereas ESC signature genes showed a negative enrichment (Fig. 2d).

To further substantiate our RNA-seq results, we performed reverse transcription coupled quantitative real-time PCR (qRT–PCR) in Gas41 shRNA KD and CRISPR-Cas9 KO cells using a selection of genes expected to be upregulated in various cell lineages. We found that depletion of Gas41 led to upregulation of genes of all lineages, including trophectoderm (*Gata3* and *Cdx2*), ectoderm (*Fgf5* and *Mash1*), mesoderm (*Meis1*) and endoderm (*Evx1* and *Msx1*; Fig. 2e and Supplementary Figure S2c), indicating that Gas41 suppresses the expression of differentiation genes in mESCs. However, the expression of the core pluripotent factors, Oct4, Sox2 and Nanog, were largely unaffected by Gas41 KD. (Supplementary Figure S2d, e).

Due to the concomitant expression of both developmental genes and core pluripotent factors, we speculate that the Gas41-depleted mESCs are likely in an intermediate state between undifferentiated and fully differentiated states, as previously observed in mESCs depleted of the Tip60/p400 complex^29^. Taken together, these results suggest that Gas41 is required for maintenance of ESC identity through transcriptional suppression of developmental genes.

### Gas41 maintains the chromatin occupancy of H2A.Z globally

**Fig. 3:**
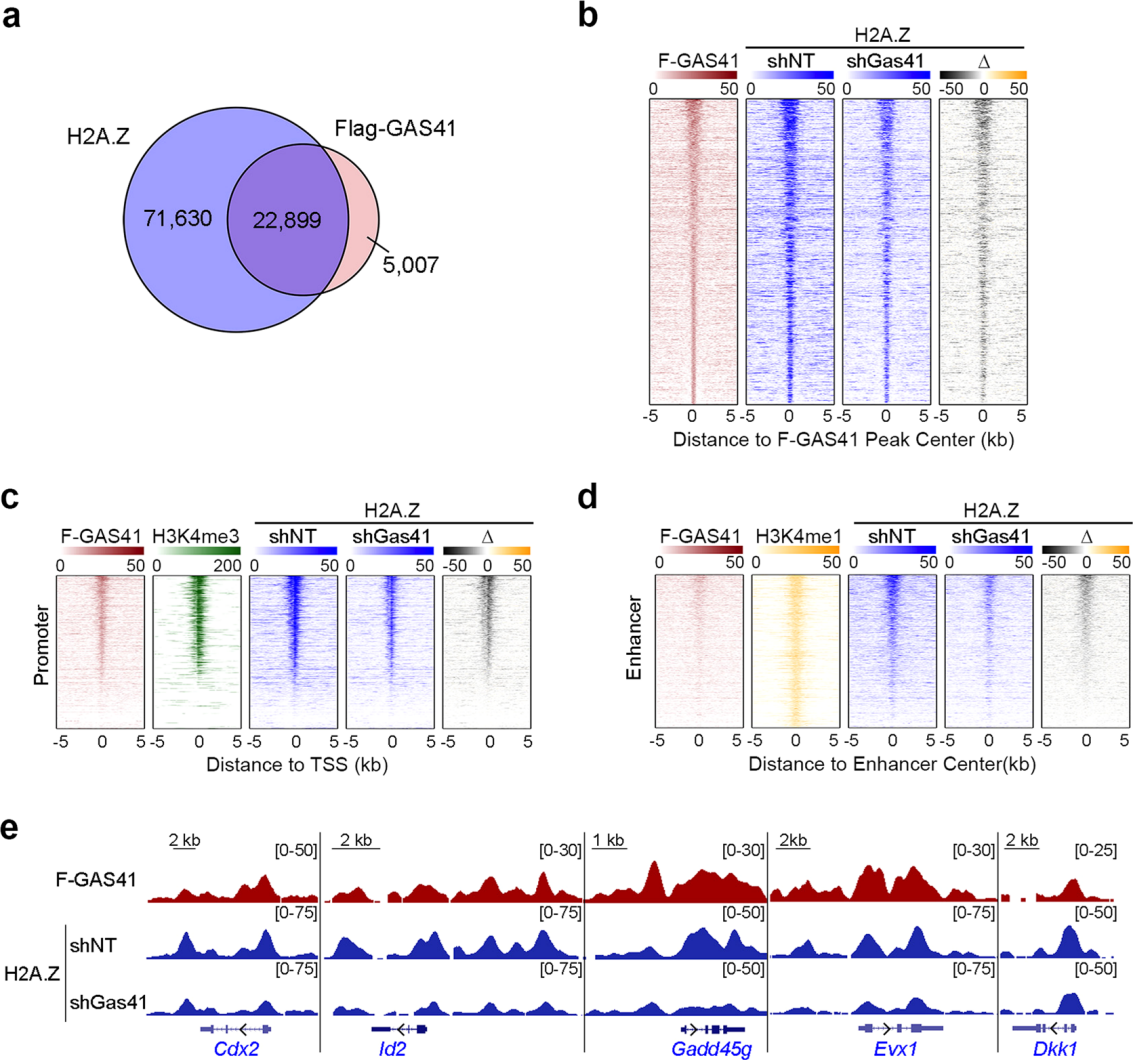
Gas41 knockdown reduces H2A.Z occupancy globally. **a** Venn diagram showing the overlap of Flag-GAS41 and H2A.Z occupied peaks. **b** Heatmap profiles of Flag-GAS41 and H2A.Z in control (shNT), Gas41 KD (shGas41) mESCs, and difference (Δ, shGas41-shNT). Gray indicates a reduction in Gas41 KD cells compared to the control cells. **c** Heatmaps profiles of Flag-GAS41 and H2A.Z occupancies at transcription start sites (TSSs) in cells as in **b**. The H3K4me3 profile in control cells are shown to define active promoters. **d** Heatmaps profiles of Flag-GAS41 and H2A.Z occupancies at enhancers in cells as in **b**. H3K4me1 ChIP-seq reads from GSM845238 is shown to define enhancers. **e** Genome browser views of the Flag-GAS41 (dark red) and H2A.Z (blue) ChIP-seq peaks on the indicated genes in cells as in **c**

Gas41 is a shared component of the Tip60/p400 and SRCAP complexes, both of which catalyze the exchange of H2A for H2A.Z on chromatin^17–19^. H2A.Z has been shown to be essential for stem cell identity maintenance^11–13^. Therefore, we asked whether Gas41 maintains stem cell identity through H2A.Z deposition. To profile genomic distribution of Gas41 and its correlation with H2A.Z occupancy, we performed chromatin immunoprecipitation (ChIP) followed by high-throughput sequencing (ChIP-seq). Extensive attempts to ChIP for endogenous Gas41 using commercial antibodies failed. Therefore, we generated mESCs stably expressing 3Flag-tagged human GAS41 (which is identical to the mouse Gas41 protein except for one amino acid at the C-terminus, Supplementary Figure S3a) and performed ChIP experiments using the M2 Flag antibody. We identified 27,906 Flag-GAS41 occupied peaks and 94,529 H2A.Z peaks (Supplementary Table S3). Strikingly, more than 80% of the Flag-GAS41 peaks overlapped with H2A.Z occupancy (Fig. 3a, b).

H2A.Z occupies both gene promoters and enhancers^11,15,16^. Similarly, we found that Flag-GAS41 was also localized on both promoters and enhancers, with a slightly higher occupancy on active promoters than enhancers (Fig. 3c, d). Importantly, Gas41 KD diminished global H2A.Z occupancy, with a greater reduction on gene promoters than enhancers (Fig. 3b–d). Furthermore, ChIP-qPCR analysis corroborated the reduction of H2A.Z occupancy on selected gene promoters (Fig. 3e and Supplementary Figure S3b).

The reduction of H2A.Z in Gas41 KD cells occurred at the protein level (Supplementary Figure S3c), as the mRNA levels of *H2afv* and *H2afz*, the two H2A.Z encoding genes, remained largely unchanged upon Gas41 KD (Supplementary Figure S3d). To determine whether reduction of H2A.Z chromatin occupancy is a cause or consequence of diminished total H2A.Z protein levels, we restored the total H2A.Z protein levels by ectopically overexpressing H2A.Z in Gas41 KD cells (Supplementary Figure S3e). However, restoration of total H2A.Z protein levels in cells did not render normal occupancy of H2A.Z at target gene promoters (Supplementary Figure S3f); and consequently, ectopic expression of H2A.Z failed to rescue the dysregulated gene expression or differentiation phenotypes of Gas41 KD cells (Supplementary Figure S3g-i). Taken together, these results suggest that Gas41 is required for maintenance of global H2A.Z occupancy on chromatin.

### Structural basis for H3K27ac recognition by the Gas41 YEATS domain

Gas41 is a small protein of about 25 kDa with only one conserved protein module, the YEATS domain. We have just recently reported that, similar to the YEATS domains of AF9 and ENL that function as readers of histone acetylation^20,21^, Gas41 YEATS also binds to acetylated histones, with a preference toward to acetylation on H3K27 and H3K14^30^. To decipher the molecular mechanism underlying H3K27ac readout by the Gas41 YEATS domain, we determined the crystal structure of Gas41 YEATS (aa 15–159) bound to a histone H3_15-39_K27ac peptide at 2.1 Å (Table 1). We found that the Gas41 YEATS domain formed four monomers in one asymmetric unit, with only one monomer occupied with peptide (Supplementary Figure S4a). Strikingly, an N-terminal extended loop produced by the tag cleavage contributed to the crystal packing by parallel ‘β strand-loop’ (β8-extended loop) formation with the adjacent asymmetric unit (Supplementary Figure S4b, c). On the basis of electron density maps, only one monomer in each asymmetric unit displayed this extended N-terminal loop, indicating that the observed crystal packing was likely induced by asymmetric protein degradation during protein purification or crystal growth. Size exclusion chromatography followed by static light scattering analysis demonstrated that the Gas41 YEATS domain was primarily monomeric in solution (Supplementary Figure S4d).

**Table 1:** Data collection and refinement statistics

	YEATS_GAS41_-H3_15-39_K27ac
*Data collection*	
Space group	P2_1_2_1_2_1_
*Cell dimensions*	
*a*, *b*, *c* (Å)	75.9, 80.7, 107.0
α, β, γ (°)	90, 90, 90
Wavelength (Å)	0.9791
Resolution (Å)	50-2.1 (2.18-2.10)^a^
*R*_merge_ (%)	7.3 (87.1)
*I* / σ*I*	16.85 (3.23)
Completeness (%)	99.6 (96.2)
Redundancy	10.1 (10.1)
*Refinement (****F*** > *0)*	
Resolution (Å)	49.1–2.1
No. reflections (test set)	38636 (3696)
*R*_work_/*R*_free_ (%)	19.3/23.2
*No. atoms*	
Protein	4706
Ligand	8
Water	148
*B-factors (Å* ^*2*^ *)*	
Protein	30.1
Ligand	22.8
Water	27.8
*R.m.s. deviations*	
Bond lengths (Å)	0.008
Bond angles (°)	1.11

^a^ Values in parentheses are for highest-resolution shell

**Fig. 4:**
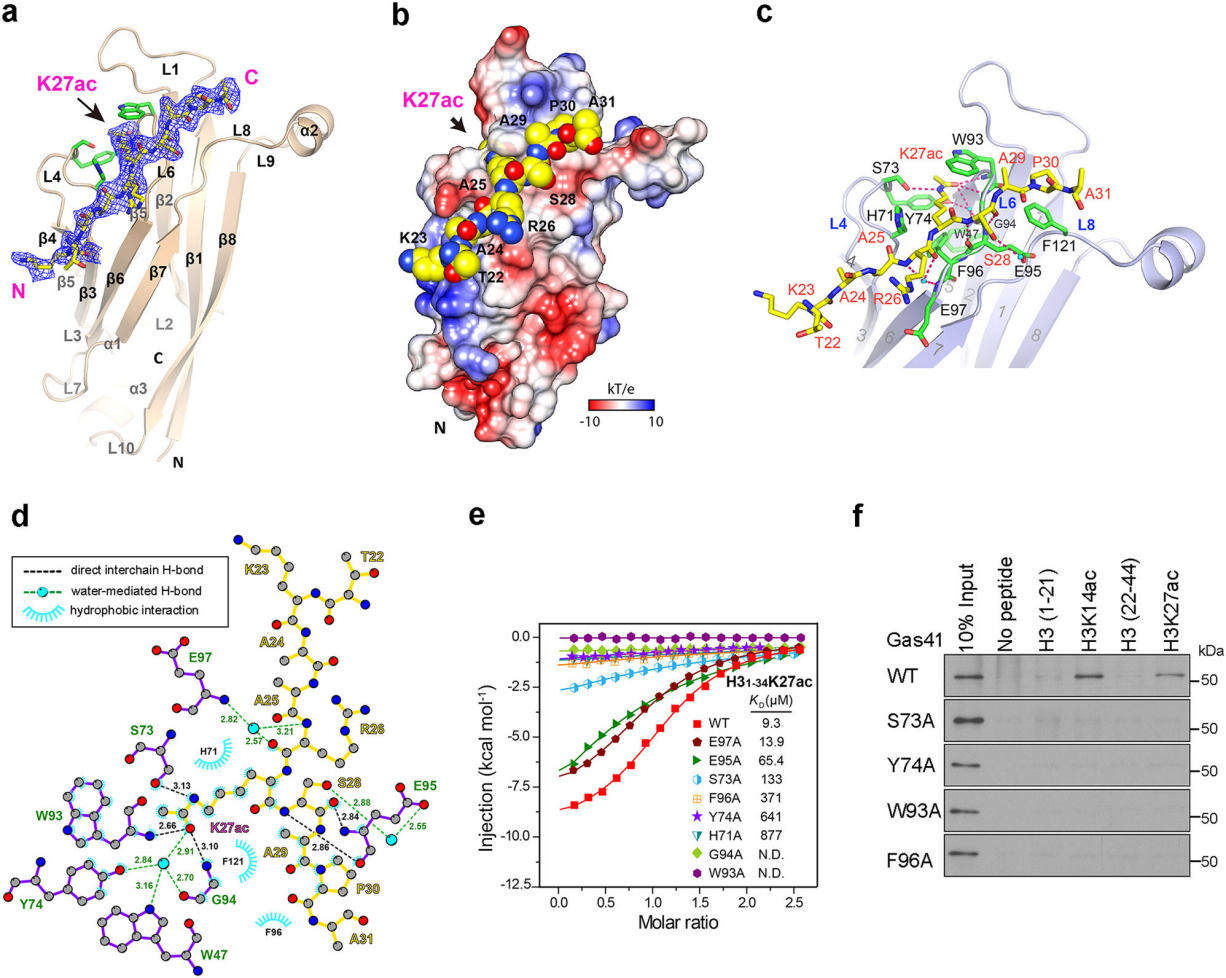
Structural basis for H3K27ac recognition by the Gas41 YEATS domain. **a** Overall structure of the Gas41 YEATS domain bound to H3 (15-39) K27ac peptide. The YEATS domain is shown as a wheat ribbon with key residues of the K27ac binding pocket depicted as a stick model (green). H3K27ac peptide is shown as yellow sticks. Dark blue mesh: Fo-Fc omit map around H3 (15-39) K27ac peptide contoured at 2.0 level. **b** Electrostatic surface view of the Gas41 YEATS domain-H3K27ac complex structure. Electrostatic potential is expressed as a spectrum ranging from −10 kT/e (red) to +10 kT/e (blue). The H3K27ac peptide is depicted as a space-filling sphere with yellow for carbon, blue for nitrogen and red for oxygen atoms. **c** Hydrogen bonding network between H3K27ac peptide and Gas41. Hydrogen bonds are shown as pink dashes. Key residues of Gas41 are depicted as a green stick model with residues labeled in black; the H3 peptide is shown as a yellow stick model with residues labeled in red. **d** LIGPLOT diagram showing critical contacts between the H3K27ac peptide and Gas41 YEATS. The H3 segment (yellow) and key residues of Gas41 YEATS (purple) are depicted in ball-and-stick mode with carbon in gray, nitrogen in dark blue, oxygen in red and water in cyan. **e** ITC fitting curves of Gas41 YEATS WT and mutants titrated with H3 (1–34) K27ac peptides. **f** Western blot analysis of histone peptide pulldowns of GST-Gas41 WT or mutants with indicated histone H3 peptides

In the structure of the complex, the Gas41 YEATS domain adopts an immunoglobin β-sandwich fold consisting of eight antiparallel β strands and three α helices connected by loop regions (Fig. 4a). The H3 binding surface of the Gas41 YEATS domain is electrostatically negatively charged, facilitating binding to the basic H3 peptide (Fig. 4b). Protein sequence alignment (Supplementary Figure S5a) combined with residue conservation analysis (Supplementary Figure S5b) of the YEATS domain of Gas41 orthologues from different species revealed that the amino acids forming the H3K27ac binding pocket are strictly conserved, suggesting that recognition of acetyllysine is an evolutionally conserved function of Gas41.

In the Gas41 YEATS-H3K27ac complex, the highly-conserved residues H71, S73, Y74 of loop 4 (L4), as well as W93 and F96 of loop 6 (L6) of Gas41 generate a serine-lined aromatic cage for acetyllysine (Kac) recognition (Fig. 4c). In addition, flanking residues of histone H3K27 also contribute to the site-specific readout by Gas41 YEATS. H3S28 participates in direct and water-mediated hydrogen bonds with Gas41 E95; and H3R26 forms a charge-stabilized interaction with E97 (Fig. 4d). In respect of the hydrogen bond networks between the H3 and Gas41 YEATS, additional hydrophobic contacts introduced by hydrocarbon chain extension (H71, G92, F96, F121) of Gas41 further stabilize the interaction (Fig. 4d).

To verify the importance of key K27ac binding residues within the Gas41 YEATS domain, we substituted alanine for several YEATS domain residues that are expected to be important for H3K27ac binding based on our crystal structure, and performed isothermal titration calorimetry (ITC) and peptide pull-down binding studies (Fig. 4e, f, and Supplementary Table S4). Replacing residues H71, S73, Y74 and F96 with alanine resulted in ~14-fold to 94-fold binding reduction. Compared with a mild binding reduction of S73A (14-fold reduction), Y74A and W93A led to the most dramatic binding loss (69-fold affinity drops for Y74A; no detected binding for W93A), highlighting the critical role of these two sandwiching residues in Kac recognition. G94 is part of the inner wall of the K27ac binding pocket and the complete binding loss of the G94A mutant reveals the necessity of a side chain-free G94 for proper Kac insertion. In addition, mutation of Gas41 E95, which directly contacts H3S28, led to a seven-fold loss of affinity.

We previously crystalized the YEATS domain of AF9 in complex with an H3K9ac peptide^20^. By comparing Gas41-H3K27ac with the AF9-H3K9ac interaction, we found that the Kac binding pockets of both YEATS domains are composed of residues with relatively high conservation (Supplementary Figure S5c). The most obvious difference between the two complex structures is the orientation of the H3 peptide. In the complex structure of AF9-H3K9ac, the N-terminal region “K4-Q5-T6-A7-R8” of H3K9 participates in AF9 binding (Supplementary Figure S5c, right panel), whereas in the case of GAS41-H3K27ac, both the N- and C-terminal flanking regions of H3K27, “A25-R26” and “S28-A29-P30”, contribute to GAS41 binding by forming hydrogen bond and hydrophobic interactions with E95, E97, F96, F121 (Fig. 4d and Supplementary Figure S5c, left panel). Therefore, although both the H3K9 and H3K27 sites contain a consensus “ARKS” motif, these distinct recognition signatures partially explain the site specificity of the GAS41 and AF9 YEATS domains towards H3K27ac or H3K9ac, respectively.

### H2A.Z co-localizes with H3K27ac and H3K14ac across the genome

**Fig. 5:**
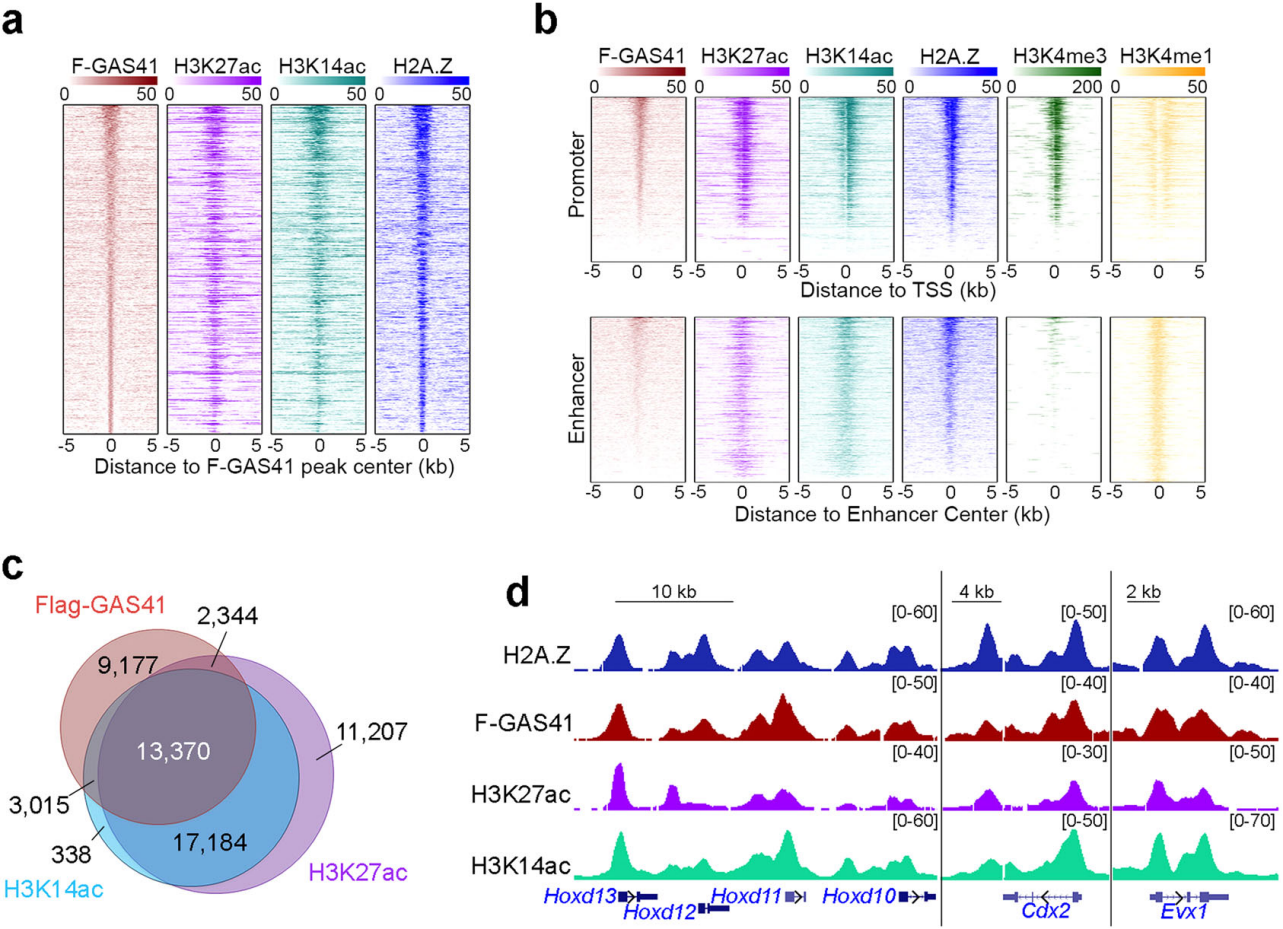
H2A.Z co-localizes with H3 acetylation across the genome. **a** Heatmap profiles of Flag-GAS41, H3K27ac, H3K14ac and H2A.Z occupancies in mESCs. **b** Heatmaps profiles of Flag-GAS41, H3K27ac, H3K14ac and H2A.Z occupancies at active promoters (upper panel) and enhancers (lower panel) in mESCs. H3K4me3 marks active promoters and H3K4me1 marks enhancers. **c** Venn diagram showing the 3-way overlaps of Flag-GAS41-, H3K27ac- and H3K14ac peaks. **d** Genome browser views of the H2A.Z (blue), Flag-GAS41 (dark red), H3K27ac (purple) and H3K14ac (cyan) ChIP-seq peaks on the indicated genes

The biochemical and structural data prompted us to determine whether Gas41 co-localizes with acetylated histone H3K14 and H3K27 in cells. We performed H3K27ac and H3K14ac ChIP-seq, which revealed 44,105 and 33,907 peaks, respectively (Supplementary Table S3). Heatmap analysis of the ChIP-seq data revealed that Flag-GAS41 bound peaks co-localized with H3K27ac and H3K14ac peaks across the genome (Fig. 5a). The co-localization was observed on both promoters and enhancers (Fig. 5b). Strikingly, over two-thirds of the Flag-GAS41-occupied genes were also enriched in H3K27ac or H3K14ac occupancy or both (Fig. 5c). Genome browser views of ChIP-seq signals showed the colocalization of Flag-GAS41 with H3K27ac, H3K14ac, and H2A.Z on gene promoters (Fig. 5d).

To determine whether the recognition of H3 acetylation by the YEATS domain is critical for GAS41 chromatin occupancy, we compared genomic distribution of Flag-tagged wild-type (WT) GAS41 and GAS41-W93A, an acetyl-binding deficient mutant, stably expressed in J1 mESCs (Supplementary Figure S6a) by ChIP-seq experiments using the M2 Flag antibody. We found that the W93A mutant exhibited diminished chromatin occupancy compared to WT GAS41 (Supplementary Figure S6b), suggesting that the YEATS domain is essential for GAS41 chromatin occupancy. Together, all these data suggest that Gas41 binds to acetylated histone H3 in the chromatin and may thus help recruit the Tip60/p400 and SRCAP complexes to acetylation-enriched regions to exchange the canonical H2A for the H2A.Z variant.

### The Gas41 YEATS domain safeguards mESC identity by modulating H2A.Z occupancy

**Fig. 6:**
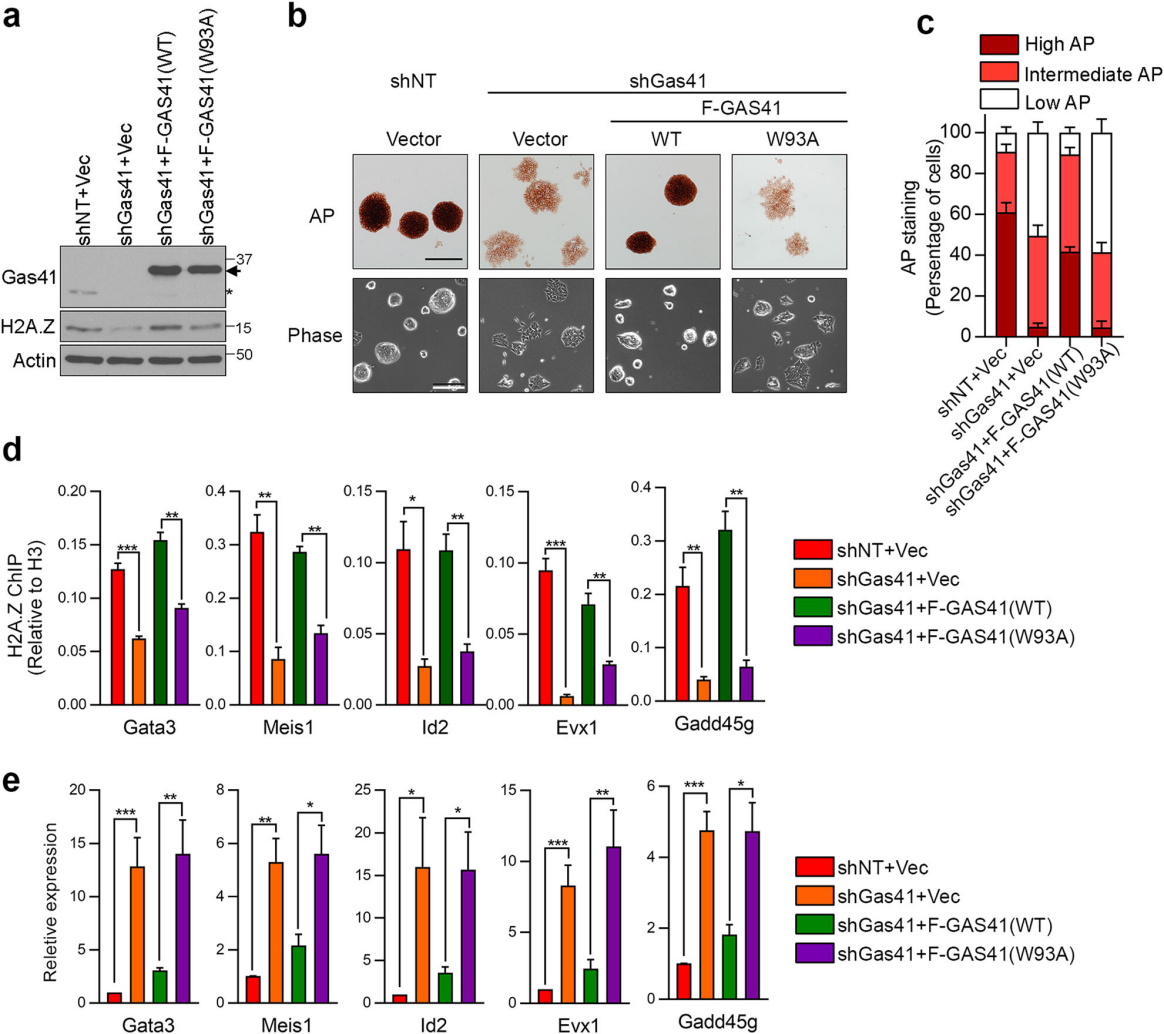
The YEATS domain of Gas41 safeguards mESC identity. **a** Western blot analysis of Gas41 and H2A.Z in control (shNT + vector), Gas41 KD (shGas41), and Gas41 KD mESCs expressing Flag-tagged WT or mutant (W93A) GAS41. Actin serves as a loading control. The arrow indicates Flag-GAS41 proteins and the asterisk denotes endogenous Gas41. **b** AP staining (upper panel) and phase contrast imaging (lower panel) of cells as in **a**. Bar, 200 μM. **c** Stacked bar graph showing quantification of AP staining from **b**. Error bars represent the SEM from 5 (shNT + Vec and shGas41 + W93A) or 6 (shGas41 + Vec and shGas41 + WT) randomly selected fields. Statistical analyses are available in Supplementary Table S7. **d** ChIP-qPCR analysis of H2A.Z occupancy at the promoters of bivalent genes in cells as in **a** Error bars represent the SEM of technical triplicates. *P < 0.05, **P < 0.01, ***P < 0.001 (Two-tailed unpaired Student’s *t*-test). Statistics source data are shown in Supplementary Table 7. **e** RT–PCR analysis of expression of the indicated bivalent genes in cells as in **a**. Error bars represent the SEM from *n* = 7 (Gata3), 6 (Evx1), and 4 (Meis1, Id2, and Gadd45g) biologically independent experiments. *P < 0.05, **P < 0.01, ***P < 0.001 (Two-tailed unpaired Student’s *t*-test). Statistics source data are shown in Supplementary Table S7

To determine whether the acetylation reader function of the Gas41 YEATS domain is essential for maintaining ESC identity, we performed rescue experiments in the Gas41 KD mESCs by ectopically expressing either Flag-tagged WT GAS41 or the W93A mutant (Fig. 6a). Expression of WT GAS41, but not W93A, fully rescued the differentiation phenotypes of the Gas41 KD cells in both AP staining and morphology assays (Fig. 6b, c), suggesting that recognition of histone acetylation by the YEATS domain is important for the function of Gas41 in mESCs.

Depletion of Gas41 led to reduction of global H2A.Z levels, and the reduction can be rescued by ectopic expression of WT GAS41 but not the W93A mutant (Fig. 6a). These results suggest that the Gas41 YEATS domain is required for H2A.Z deposition in mESCs. To test this hypothesis, we performed ChIP experiments in the Gas41 KD and rescued mESCs to determine H2A.Z promoter occupancy at selected developmental genes. ChIP-qPCR analysis revealed that the reduction of H2A.Z promoter occupancy in Gas41 KD cells was rescued by WT GAS41, but not the W93A mutant (Fig. 6d). Consequently, the elevated expression of these developmental genes in Gas41 KD cells was counteracted by the WT GAS41, but not the mutant (Fig. 6e). Taken together, these results suggest that the Gas41 YEATS domain recognizes acetylated histone H3 and facilitates H2A.Z deposition on chromatin, and thus safeguards mESC identity by preventing premature activation of developmental genes.

### Gas41 maintains a subset of bivalent domains in the poised state

**Fig. 7:**
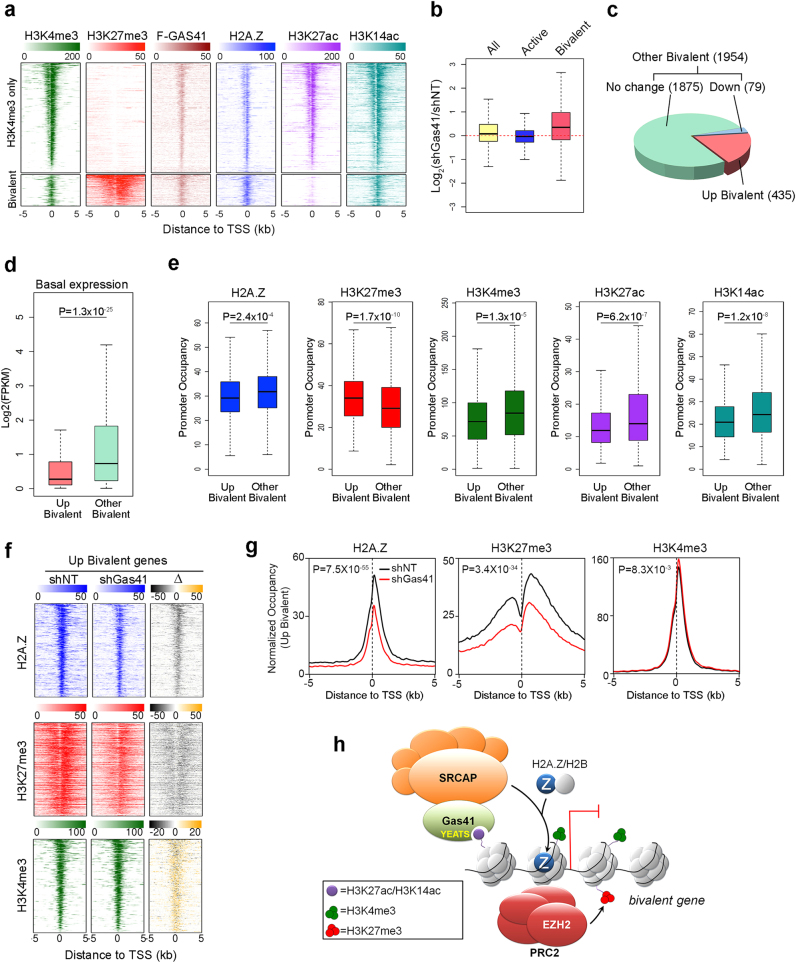
Gas41 KD perturbs the balance of bivalent domains. **a** Heatmap profiles of H3K4me3, H3K27me3, Flag-GAS41, H2A.Z, H3K27ac and H3K14ac occupancies at TSSs (±5 kb) of the active (H3K4me3 only, *n* = 8618) or bivalent genes (n = 2389) in mESCs. **b** Box plots representing the log_2_ fold change in expression (FPKM, reads per kilobase per million) of Gas41 KD (shGas41-1) relative to control (shNT) mESCs at all (*n* = 19,548), active and bivalent genes. The center line represents the median and box limits indicate the 25th and 75th percentiles. **c** Pie chart showing the proportion of bivalent genes that are upregulated (Up Bivalent, log_2_FC ≥ 1, FDR ≤ 0.05), downregulated (Down, log_2_FC ≤ −1, FDR ≤ 0.05), or genes with no change upon Gas41 KD. The “Down” and “No Change” groups were combined as “Other Bivalent”. The numbers of genes in each group are indicate in brackets. **d** Box plots showing the basal expression levels of the Up Bivalent and the Other Bivalent genes in mESCs. Center lines represent median values, and box limits indicate the 25th and 75th percentiles. Two-tailed unpaired Student’s *t*-test was used for statistical analyses. **e** Box plots of H2A.Z, H3K27me3, H3K4me3, H3K27ac, and H3K14ac occupancies at promoters of the Up Bivalent genes or Other Bivalent genes in mESCs. Boxes are defined as in **b**. Two-tailed unpaired Student’s t-test was used for statistical analyses. **f** Heatmap profiles of H2A.Z, H3K27me3 and H3K4me3 occupancies at the TSSs (±5 kb) of the Up Bivalent genes in control (shNT), Gas41 KD (shGas41) mESCs, and the difference between them (Δ, shGas41-shNT). Gray indicates a reduction and yellow indicates an increase in the Gas41 KD cells compared to the control cells. **g** Average profiles of H2A.Z (left panel), H3K27me3 (middle panel) and H3K4me3 (right panel) occupancies at the TSS (±5 kb) of the Up Bivalent genes in control (shNT, black line) and Gas41 KD (shGas41, red line) mESCs. Significance is determined by two-tailed unpaired Wilcoxon test. **h** Working model. Gas41 binds to acetylation on H3K27 and H3K14 through its YEATS domain and facilitates the recruitment of the SRCAP complex to bivalent gene promoters to exchange the canonical H2A for H2A.Z, which, in turn, maintains a poised state of the chromatin by facilitating the recruitment of PRC2 complex

Next, we sought to determine whether Gas41 regulates a specific set of genes in maintaining the ESC phenotypes. As many of the developmental genes are epigenetically bivalent containing both H3K4me3 and H3K27me3 marks^5,6^, we first compared Gas41 occupancy on the bivalent genes vs active genes that contain only H3K4me3. We found that Flag-GAS41 occupies both bivalent and active promoters with a slightly stronger occupancy on bivalent genes, a distribution pattern similar to that of H2A.Z (Fig. 7a). Importantly, the bivalent genes exhibited the greatest changes in gene upregulation upon Gas41 KD (Fig. 7b), suggesting that Gas41 may preferentially suppress the bivalent genes in mESCs. In supporting this, GSEA analysis revealed an enrichment of bivalent genes of the genes upregulated in Gas41 KD cells (Supplementary Figure S6c).

Among the 2389 bivalent genes we identified in mESCs (defined by enrichment of both H3K4me3 and H3K27me3 ChIP-seq signals, Supplementary Table S5), 435 were upregulated (fold change ≥2) in Gas41-depleted cells, and 79 were downregulated (Fig. 7c and Supplementary Table S5). For simplicity, we divided all these bivalent genes into two categories, “Up Bivalent” and “Other Bivalent”, as inclusion of the minor subset of downregulated genes did not affect the overall analysis. We first assessed the basal expression levels of these two groups of genes in mESCs. Interestingly, we found that in undifferentiated mESCs, the averaged basal expression levels of the Up Bivalent genes were significantly lower than the Other Bivalent genes (*P* = 1.3 × 10^−25^; Fig. 7d and Supplementary Figure S6d), indicating that Gas41 preferentially suppresses bivalent genes with lower expression levels in mESCs. Consistent with gene expression, the Up Bivalent genes exhibited significantly higher levels of H3K27me3 and lower levels of H3K4me3, H3K27ac and H3K14ac (Fig. 7e). However, surprisingly, although H2A.Z was shown to have a repressive role on bivalent genes^14,31^, we observed slightly lower levels of H2A.Z levels on the low-expressing Up Bivalent genes compared to the Other Bivalent genes.

To understand how Gas41 depletion derepresses bivalent gene expression, we determined changes in H2A.Z and the bivalent marks (H3K4me3 and H3K27me3) at promoters of the Up Bivalent genes upon Gas41 KD. Consistent with our observation of global H2A.Z changes, Gas41 KD reduced H2A.Z levels on the Up Bivalent genes (Fig. 7f, g). Concomitantly, the repressive mark H3K27me3 was also reduced whereas the active mark H3K4me3 slightly increased on the Up Bivalent genes upon Gas41 KD (Fig. 7f, g and Supplementary Figure S6e). By ChIP-qPCR analysis, we also observed perturbations in the balance of bivalent domains at the individual bivalent genes upon depletion of Gas41 (Supplementary Figure S6f-h), leading to derepression of gene expression (Supplementary Figure S6i).

Gas41 is a shared component Tip60/p400 and SRCAP complexes. To determine which complex is responsible for Gas41 function in mESCs, we KD Ep400 and Znhit1, which are Tip60/p400 and SRCAP complex specific subunit, respectively, and assessed global H2A.Z levels and cellular phenotypic changes. Both KDs resulted in flattened morphology and reduced AP staining intensity of mESCs (Supplementary Figure S7a-c), however, only Znhit1 KD led to reduction in global H2A.Z protein levels (Supplementary Figure S7d). Next, we next determined genomic distribution of Znhit1 by ChIP-seq experiments in Flag-ZNHIT1 expressing J1 cells, and compared to the chromatin occupancy of Gas41 and p400^32^. The results revealed that ZNHIT1 co-localizes with GAS41 in both active and bivalent promoters whereas p400 is mainly enriched in active promoters; and consistent with their genomic occupancy, depletion of Znhit1, but not p400, diminished H2A.Z occupancy on both active and bivalent promoters (Supplementary Figure S7e). Taken together, these results suggest the Gas41 promotes H2A.Z deposition at bivalent promoters mainly through the SRCAP complex.

In summary, our findings support a model in which Gas41 recognizes histone H3K27 and H3K14 acetylation and facilitates the SRCAP complex-dependent deposition of H2A.Z, which in turn helps to maintain bivalent domains in a transcriptionally poised state in mESCs (Fig. 7h).

## Discussion

Acetylation is one of the most common PTMs on histones. In addition to help opening up chromatin by reducing the charge interaction between histones and DNA, acetylation provides a binding platform for many reader proteins, which in turn recruit a variety of histone-modifying and chromatin remodeling complexes^33,34^. Until the recent identification of the YEATS domain as a new family of acetylation readers^20^, the bromodomains were thought to be the only acetylation readers. The YEATS domains differ from the bromodomains in that they are specific for histone H3 lysine acetylation, with no detectable binding of the YEATS domains to other acetylated histones. Both the AF9 and ENL YEATS domains recognize acetylated lysine following arginine within the “RK” motif, which explains their specific recognition of acetylation on H3K9, H3K18, and H3K27^20,21^.

Gas41 shares the same mode of acetyllysine recognition as AF9 and ENL with respect to H3K27ac. However, the Gas41 YEATS domain is unique in that it can also bind to acetylation on H3K14, which lacks the “RK” motif. Although the mechanism of H3K14ac recognition is currently unknown due to the lack of a crystal structure, this unique YEATS domain binding specificity indicates that Gas41 may carry out specialized biological functions. Intriguingly, in contrast to H3K9ac and H3K27ac that are tightly associated with active promoters and enhancers, H3K14ac is also enriched at a subset of inactive bivalent promoters in mESCs^35^, suggesting that this specialized Gas41 function might be related to a role at bivalent promoters. In line with this, we find that depletion of Gas41 selectively derepresses a subset of bivalent genes with low gene expression. As the histone H3K27 residue within the bivalent domains is mostly marked with methylation thus unavailable for acetylation, it is likely that acetylation on H3K14 plays a dominant role in recruiting Gas41 to bivalent promoters in mESCs. Prior studies have shown that H2A.Z is preferentially deposited into nucleosome with high level of histone H4 acetylation^36,37^. We find that GAS41 is also able to bind to acetylated H4 peptides, albeit much weaker than its binding to the H3K27ac or H3K14ac peptide. Further studies are needed to determine whether the weak binding to H4 acetylation by the YEATS domain contributes to the chromatin recruitment of GAS41 and the associated chromatin remodeling complexes.

H2A.Z plays both a positive and a negative role in the regulation of gene expression. In mESCs, H2A.Z occupies active (H3K4me3 only) as well as bivalent promoters^11,15^, where it strongly colocalizes with the PRC2 complex^14^. The dual function of H2A.Z has been proposed to be a consequence of distinct PTMs on this variant histone, with acetylation promoting gene activation^11,15,38,39^ and monoubiquitylation contributing to gene repression^31,40^. In bivalent promoters, H2A.Z is enriched with monoubiquitylation, which facilitates the recruitment of the PRC2 complex and repels the association of the BRD2 transcriptional coactivator on bivalent domains, thus helping to maintain these genes in a poised state^15,31^. Consistent with these observations, we find that Gas41 depletion leads to concomitant reduction of both H2A.Z and H3K27me3 occupancies at bivalent promoters.

Although H2A.Z occupancy is reduced on almost all bivalent genes upon Gas41 KD, only ~20% exhibit a ≥two-fold induction of gene expression. Compared to the bivalent genes whose expression is not altered by Gas41 KD, the derepressed bivalent genes have lower basal expression levels in the undifferentiated mESCs. It is tempting to speculate that for the unaffected bivalent genes, some other mechanisms may compensate H2A.Z loss. It will be interesting to determine in future studies whether the H2A.Z-independent bivalent genes have, or lack, some epigenetic characters that are distinct from the H2A.Z-dependent bivalent genes.

The Tip60/p400 complex has been previously shown to be required for maintaining ESC identity^29^. P400 is predominantly involved in transcriptional repression of the bivalent genes^32^. Therefore, preventing bivalent genes from faulty activation is likely a major means by which the Tip60/p400 complex keeps ESCs undifferentiated. Interestingly, despite its repressive role toward bivalent genes, p400 preferentially binds active gene (H3K4me3 only) than bivalent genes^32^. Moreover, p400 KD exhibited minimal effect on H2A.Z occupancy at bivalent promoters. Therefore, it is likely that Tip60/p400 complex suppress bivalent gene expression through mechanisms other than H2A.Z deposition. On the contrary, we find the SRCAP complex co-localizes with GAS41 at both active and bivalent genes, and depletion of Znhit1 leads to reduction of H2A.Z occupancy at bivalent promoter, suggesting that the SRCAP complex plays a major role for Gas41-mediated H2A.Z deposition in mESCs.

In summary, our study unravels an essential role of the Gas41 YEATS domain by linking histone acetylation to H2A.Z deposition and the maintenance of ESC identity. In addition to ESC self-renewal, H2A.Z was also shown to be essential for ESC differentiation^11,14^. In the future, it will be of great interest to determine whether Gas41 and its YEATS domain also play a role in modulating ESC lineage commitment.

## Methods

### Materials

Human Gas41 cDNAs were cloned into pENTR3C and subsequently cloned into pHAGE-3xFlag destination vectors using Gateway techniques (Invitrogen). Point mutations were generated using a site-directed mutagenesis kit (Stratagene). Histone peptides bearing different modifications were synthesized by either the W.M. Keck Facility at Yale University or SciLight Biotechnology, LLC. All pLKO shRNA constructs were purchased from Sigma. sgRNAs were cloned into lentiCRISPR v2 (Addgene Plasmid #52961). Information of all oligoes, antibodies and shRNAs used are provided in Supplementary Table S6.

### Protein production

The gene of human GAS41 was amplified from HEK 293 cDNA library and verified by sequencing. The YEATS domain encompassing residues 15–159 of GAS41 was cloned in to a pET28b vector. The recombinant GAS41_15-159_ was overexpressed in *Escherichia coli* strain BL21 (DE3).

After overnight induction by 0.4 mM isopropyl β-D-thiogalactoside (IPTG) at 16 °C in TB medium, cells were harvested and resuspended in buffer, 20 mM Tris-HCl, pH 7.5, 500 mM NaCl, 5% glycerol, 1 mM phenylmethylsulfonyl fluoride (PMSF) and 20 mM imidazole. After cell lysis and centrifugation, the recombinant protein was purified to homogeneity over a HisTrap column, and the 6× His tag was cleaved by thrombin overnight at 4 °C. The released GAS41_15–159_ protein was further purified by size exclusion chromatography using a Superdex G75 column (GE Healthcare) in elution buffer (20 mM Tris-HCl, pH 7.5, 500 mM NaCl, 5% glycerol and 2 mM β-mercaptoethanol). All GAS41_15–159_ mutants were generated using QuikChange site-directed mutagenesis kit (Stratagene) and verified by sequencing. The purification procedures for the GAS41 YEATS mutants were essentially the same as for the wild-type protein.

### Crystallization, data collection, and structure determination

Crystallization was performed via the sitting drop vapor diffusion method under 18 °C by mixing equal volumes (0.8 μl) of GAS41 YEATS-H3_15-39_K27ac (1:2 molar ratio, 7 mg/ml) and reservoir solutions: 0.1 M sodium acetate trihydrate, pH 4.6, 4.0 M ammonium acetate. The complex crystals were briefly soaked in cryoprotectant containing the reservoir solution supplemented with 30% glycerol and then flash frozen in liquid nitrogen for data collection. The diffraction data set was collected at the beamline BL17U of the Shanghai Synchrotron Radiation Facility at 0.9791 Å. All diffraction images were indexed, integrated, and merged using HKL2000^41^. The structure was determined by molecular replacement using MOLREP^42^ using the AF9-H3K9ac structure (PDB ID: 4TMP) as the search model. Structural refinement was carried out using PHENIX^43^, and iterative model building was performed with COOT^44^. Detailed data collection and refinement statistics are summarized in Table 1. Structural Figures were created using PYMOL (http://www.pymol.org/).

### Isothermal titration calorimetry (ITC)

All calorimetric experiments involving wild-type or mutant GAS41 YEATS domain proteins were conducted at 15 °C using a MicroCal ITC200 instrument (GE Healthcare). The GAS41 (15–159) samples were dialyzed in the following buffer: 20 mM Tris-HCl, pH 7.5, 500 mM NaCl, 5% glycerol and 2 mM β-mercaptoethanol. Protein concentrations were determined by absorbance spectroscopy at 280 nm. Peptides (>95% purity) were quantified by weight, prior to aliquoting and freeze-drying for individual use. Calorimetric titration curves were analyzed using Origin 7.0 (OriginLab) and the “One Set of Binding Sites” fitting model. Detailed thermodynamic parameters of each titration are summarized in Supplementary Table S4.

### Peptide microarray and peptide pull-down assays

Peptide microarray and was performed as described previously^45^. In brief, biotinylated histone peptides were printed in triplicate onto a streptavidin-coated slide (PolyAn) using a VersArray Compact Microarrayer (Bio-Rad). After a short blocking with biotin (Sigma), the slides were incubated with the glutathione S-transferase (GST)-tagged GAS41 in binding buffer (50 mM Tris-HCl, pH 7.5, 250 mM NaCl, 0.1% NP-40, 1 mM PMSF, 20% FBS) overnight at 4 °C with gentle agitation. After being washed with the same buffer, the slides were probed with an anti-GST primary antibody and then a fluorescein-conjugated secondary antibody and visualized using a GenePix 4000 scanner (Molecular Devices).

For peptide pulldowns, 1 µg of biotinylated histone peptides with different modifications were incubated with 1–2 µg of GST-fused WT GAS41 or indicated mutants in binding buffer (50 mM Tris-HCl 7.5, 250 mM NaCl, 0.1% NP-40, 1 mM PMSF) overnight. Streptavidin beads (Amersham) were added to the mixture, and the mixture was incubated for 1 h with rotation. The beads were then washed three times and analyzed using SDS-PAGE and Western blotting.

### Size exclusion chromatography with multiangle light scattering

For size exclusion chromatography with multiangle light scattering, GAS41 YEATS (100 μl at 2 mg/ml in buffer containing 20 mM Tris-HCl, pH 7.5, 500 mM NaCl, 2% glycerol) was fractionated on a WTC-030S5 size exclusion column (Wyatt Technology) coupled to an AKTA Purifier 10 system (GE Healthcare) at a flow rate of 0.5 ml/min. The molecular species separated by the column were then analyzed using a Wyatt Dawn Heleos II multiangle light scattering device. The molecular mass was calculated using the Astra 6 software program (Wyatt Technology).

### Cell culture and lentivirus infection

Mouse J1 ES cells (mESCs) were maintained without feeders in cell culture dishes coated with 0.1% (w/v) gelatin (Sigma) in ESC media (Dulbecco’s modified Eagle’s medium (DMEM, Cellgro) supplemented with 15% Stasis™ Stem Cell Qualified Fetal Bovine Serum (Gemini), 100 U/ml penicillin, 100 μg/ml streptomycin, 292 μg/ml l-glutamine, 0.1 mM β-mercaptoethanol and 1000 U/mL leukemia inhibitory factor (LIF, ESGRO, Millipore, USA)). HEK 293 T cells were cultured in DMEM supplemented with 10% fetal bovine serum (Sigma).

For lentivirus production, HEK 293 T cells in 10-cm culture dish were transiently co-transfected with 2 μg of pMD2.G, 4 μg of pPAX2 (Addgene) and 2 μg of pLKO shRNA, lentiCRISPR v2-sgRNA or pHAGE-cDNA constructs using X-tremeGENE™ HP DNA Transfection Reagent (Roche). Media were replaced with ESC media around 14 h after transfection and incubated for an additional 48 h before viral supernatants were harvested. The harvested supernatants were then filtered through a 0.45 μm PVDF filter. For infections, mESCs cells were incubated with viral supernatants in the presence of 8 µg/ml polybrene for 14 h. After 48 h, the infected cells were selected with puromycin (1 μg/ml) or blasticidin (5 μg/ml) for knockdown or overexpression experiments, respectively. All KD or KO samples were harvested 8 days after infection with shRNA viruses. For rescue experiments, stable mESCs cells with or without overexpressed 3XFlag-human GAS41 (WT or mutants) were generated followed by shRNA-mediated knockdown of endogenous Gas41.

### Embryoid body (EB) formation and retinoic acid (RA) treatment

mESCs were plated in 96-well ultra-low attachment round bottom plates (Corning) at 1000 cells/well in ES cell medium without LIF. Media was changed every other day before day 8 and daily thereafter. For RA treatment, mESCs seeded on gelatin-coated dishes were treated with 1 μM RA in ES cell medium without LIF. Undifferentiated mESCs cultured with LIF in gelatin-coated dishes, are considered as EB and RA day 0.

### Isolation of mouse embryonic fibroblasts (MEFs)

Mouse embryonic fibroblasts (MEFs) were isolated from a C57BL/6 wild-type embryo on dpc13.5 following the protocol from the Jacks lab at MIT (https://jacks-lab.mit.edu/protocols/making_mefs). The second passage of MEFs were collected for protein and mRNA isolation.

### Alkaline phosphate (AP) staining

Alkaline phosphate (AP) staining was performed using an AP Staining Kit from System Biosciences (Cat#AP100R-1) according to the user manual. mESCs were seeded at 50,000 cells/well on a gelatin-coated six-well plate for 3 days before AP staining. All AP staining was conducted 8 days following infection with shRNA viruses. For quantification, the staining intensity of each individual colony was measured using Image J. To minimize staining variation between independent experiments, the cutoff values to categorize AP intensities were set according to the corresponding shNT control cells of the same experiment. We defined the top 60%, middle 30% and bottom 10% staining intensities of the control cells as high, intermediate and low, respectively.

### ChIP and ChIP-seq analysis

ChIP analysis was performed as described previously^20^ with minor modifications. In brief, cells were cross-linked with 1% formaldehyde for 10 min. The cross-linking reaction was stopped by adding glycine to 125 mM and incubating for 5 min. Fixed cells were scraped from dishes and washed 2× with PBS. Cell pellets were resuspended in cell lysis buffer (5 mM PIEPES pH 8.0, 85 mM KCl, 1% NP-40) containing the complete protease inhibitor cocktail (Roche) and 1 mM PMSF. After 20 min rotation in 4 °C, cell nuclei were harvested by centrifugation and resuspended in nuclei lysis buffer (50 mM Tris-HCl pH 8.0, 10 mM EDTA, 1% SDS) containing protease inhibitors prior to sonication with a Bioruptor Sonicator (Diagenode) to a length of approximately 100-400 bp. Sonicated chromatin samples containing approximately 20-30 μg of DNA were diluted 4-fold with dilution buffer (20 mM Tris-HCl pH 8.0, 150 mM NaCl, 1 mM EDTA, 1% Triton X-100, 0.01% SDS) and were immunoprecipitated overnight at 4 °C with 2–3 μg of antibodies. Protein A/G beads (Millipore) were added and samples were incubated for another 2 h. The immunoprecipitates were washed 2× with low-salt buffer (20 mM Tris-HCl pH 8.0, 150 mM NaCl, 2 mM EDTA, 1% Triton X-100, 0.01% SDS), 2× with high-salt buffer (20 mM Tris-HCl pH 8.0, 500 mM NaCl, 2 mM EDTA, 1% Triton X-100, 0.01% SDS), and 1× with LiCl buffer (20 mM Tris-HCl pH 8.0, 250 mM LiCl, 1 mM EDTA, 1% NP40, 1% Na-Deoxycholate). DNA was eluted in elution buffer (50 mM NaHCO3, 1% SDS) by vigorously shaking for 15 min. Crosslinks were reversed in the presence of 0.3 M NaCl at 67 °C for 12 h. DNA was purified using a PCR purification kit (Qiagen) and analyzed by real-time PCR on an ABI 7500-FAST System using the Power SYBR Green PCR Master Mix (Applied Biosystems). Primers used in this study are listed in Supplementary Table S6.

ChIP-seq samples were sequenced on an Illumina Solexa Hiseq 2000. The raw reads were mapped to the mouse reference genome NCBI build 37 (mm9) using bowtie v1.1.0, allowing up to two mismatches. The genome ChIP-seq profiles were generated using MACS 1.4.2 with only uniquely mapped reads^45^. Clonal reads were automatically removed by MACS. The ChIP-seq profiles were normalized to 20,000,000 total tag numbers, and peaks were called at *P* ≤ 1 × 10^−8^. Gene promoters are defined as 2 kb regions centered at transcriptional stating sites (TSSs). The normalized average ChIP occupancy at promoters are used for differential analysis. For overlap analysis, peaks of different marks are merged as consensus regions and peaks that have >500 bp overlap with the consensus regions are defined as overlapped peaks.

### RNA extraction, reverse transcription, real-time PCR, and RNA sequencing analysis

Total RNA extraction, reverse transcription and quantitative real-time PCR (qPCR) were performed as previously described^20^. Gene expression values were calculated by normalization to *Actb* using the comparative CT method. Primers used in the study are listed in Supplementary Table S6.

RNA was isolated from J1 cells using the RNeasy Mini Kit (Qiagen) and prepared as instructed by the manufacturer using the TruSeq RNA Sample Preparation Kit v2 (Illumina). RNA-seq samples were sequenced using an Illumina NextSeq 500. Raw reads were mapped to the mouse reference genome (mm9) and mouse transcriptome using the TopHat2 package^46^. Transcript abundance was quantified using the htseq-count script in the Python package HTSeq v0.6.0^47^. Differentially expressed genes were determined using the edgeR Bioconductor package^48^. Genes with at least a two-fold change and FDR ≤ 0.05 were selected as differentially expressed genes. GO analysis was performed at Gene Ontology Consortium website (http://www.geneontology.org/).

### Statistical analysis

All experiments were performed with two or more biological repeats. For statistical analysis, no statistical method was used to predetermine sample size. The experiments were not randomized and investigators were not blinded. Experimental data are presented as means ± standard error of mean (SEM). Unless stated otherwise, statistical significance was calculated using a two-tailed unpaired t-test with a cutoff of *P* < 0.05 using Prism 7.03. Statistical significance levels are denoted as follows: *P < 0.05; **P < 0.01; ***P < 0.001; ****P < 0.0001. All images for AP staining were randomly selected for quantification. For box plots (Fig. 7b, e and Supplementary Figure S6d), center lines represent median values, and box limits indicate the 25th and 75th percentiles. For average profiles of ChIP-seq occupancy (Fig. 7g), significance was determined by two-tailed unpaired Wilcoxon test using R program.

### Accession numbers

Structural data have been deposited in the Protein Data Bank with the accession numbers of 5XTZ. The ChIP-seq and RNA-seq data have been deposited in the GEO database (GSE100460).

## Electronic supplementary material

Supplementary Information

Supplementary Table S1

Supplementary Table S2

Supplementary Table S3

Supplementary Table S5

Supplementary Table S6

Supplementary Table S7

## References

[1] Solter D (2006). From teratocarcinomas to embryonic stem cells and beyond: a history of embryonic stem cell research. Nat. Rev. Genet..

[2] Young RA (2011). Control of the embryonic stem cell state. Cell.

[3] Ho L, Crabtree GR (2010). Chromatin remodelling during development. Nature.

[4] Orkin SH, Hochedlinger K (2011). Chromatin connections to pluripotency and cellular reprogramming. Cell.

[5] Bernstein BE (2006). A bivalent chromatin structure marks key developmental genes in embryonic stem cells. Cell.

[6] Azuara V (2006). Chromatin signatures of pluripotent cell lines. Nat. Cell Biol..

[7] Voigt P, Tee WW, Reinberg D (2013). A double take on bivalent promoters. Genes Dev..

[8] Dryhurst D (2009). Characterization of the histone H2A.Z-1 and H2A.Z-2 isoforms in vertebrates. BMC Biol..

[9] Matsuda R (2010). Identification and characterization of the two isoforms of the vertebrate H2A.Z histone variant. Nucleic Acids Res..

[10] Faast R (2001). Histone variant H2A.Z is required for early mammalian development. Curr. Biol..

[11] Hu G (2013). H2A.Z facilitates access of active and repressive complexes to chromatin in embryonic stem cell self-renewal and differentiation. Cell Stem Cell.

[12] Li Z (2012). Foxa2 and H2A.Z mediate nucleosome depletion during embryonic stem cell differentiation. Cell.

[13] Pandey R, Dou Y (2013). H2A.Z sets the stage in ESCs. Cell Stem Cell.

[14] Creyghton MP (2008). H2AZ is enriched at polycomb complex target genes in ES cells and is necessary for lineage commitment. Cell.

[15] Ku M (2012). H2A.Z landscapes and dual modifications in pluripotent and multipotent stem cells underlie complex genome regulatory functions. Genome Biol..

[16] Barski A (2007). High-resolution profiling of histone methylations in the human genome. Cell.

[17] Ruhl DD (2006). Purification of a human SRCAP complex that remodels chromatin by incorporating the histone variant H2A.Z into nucleosomes. Biochemistry.

[18] Gevry N, Chan HM, Laflamme L, Livingston DM, Gaudreau L (2007). p21 transcription is regulated by differential localization of histone H2A.Z. Genes Dev..

[19] Wong MM, Cox LK, Chrivia JC (2007). The chromatin remodeling protein, SRCAP, is critical for deposition of the histone variant H2A.Z at promoters. J. Biol. Chem..

[20] Li Y (2014). AF9 YEATS domain links histone acetylation to DOT1L-mediated H3K79 methylation. Cell.

[21] Wan L (2017). ENL links histone acetylation to oncogenic gene expression in acute myeloid leukaemia. Nature.

[22] Okamoto K (1990). A novel octamer binding transcription factor is differentially expressed in mouse embryonic cells. Cell.

[23] Feldman N (2006). G9a-mediated irreversible epigenetic inactivation of Oct-3/4 during early embryogenesis. Nat. Cell Biol..

[24] Kuroda T (2005). Octamer and Sox elements are required for transcriptional cis regulation of Nanog gene expression. Mol. Cell. Biol..

[25] Palmieri SL, Peter W, Hess H, Scholer HR (1994). Oct-4 transcription factor is differentially expressed in the mouse embryo during establishment of the first two extraembryonic cell lineages involved in implantation. Dev. Biol..

[26] Tomioka M (2002). Identification of Sox-2 regulatory region which is under the control of Oct-3/4-Sox-2 complex. Nucleic Acids Res..

[27] Ashburner M (2000). Gene ontology: tool for the unification of biology. The Gene Ontology Consortium. Nat. Genet..

[28] Huang da W, Sherman BT, Lempicki RA (2009). Bioinformatics enrichment tools: paths toward the comprehensive functional analysis of large gene lists. Nucleic Acids Res..

[29] Fazzio TG, Huff JT, Panning B (2008). An RNAi screen of chromatin proteins identifies Tip60-p400 as a regulator of embryonic stem cell identity. Cell.

[30] Hsu CC (2018). Recognition of histone acetylation by the GAS41 YEATS domain promotes H2A.Z deposition in non-small cell lung cancer. Genes Dev..

[31] Surface LE (2016). H2A.Z.1 monoubiquitylation antagonizes BRD2 to maintain poised chromatin in ESCs. Cell Rep..

[32] de Dieuleveult M (2016). Genome-wide nucleosome specificity and function of chromatin remodellers in ES cells. Nature.

[33] Gong F, Chiu LY, Miller KM (2016). Acetylation reader proteins: linking acetylation signaling to genome maintenance and cancer. PLoS. Genet..

[34] Marmorstein R, Zhou MM (2014). Writers and readers of histone acetylation: structure, mechanism, and inhibition. Cold Spring Harb. Perspect. Biol..

[35] Karmodiya K, Krebs AR, Oulad-Abdelghani M, Kimura H, Tora L (2012). H3K9 and H3K14 acetylation co-occur at many gene regulatory elements, while H3K14ac marks a subset of inactive inducible promoters in mouse embryonic stem cells. BMC Genomics.

[36] Draker R (2012). A combination of H2A.Z and H4 acetylation recruits Brd2 to chromatin during transcriptional activation. PLoS Genet..

[37] Vardabasso C (2015). Histone variant H2A.Z.2 mediates proliferation and drug sensitivity of malignant melanoma. Mol. Cell.

[38] Bruce K (2005). The replacement histone H2A.Z in a hyperacetylated form is a feature of active genes in the chicken. Nucleic Acids Res..

[39] Millar CB, Xu F, Zhang K, Grunstein M (2006). Acetylation of H2AZ Lys 14 is associated with genome-wide gene activity in yeast. Genes Dev..

[40] Sarcinella E, Zuzarte PC, Lau PN, Draker R, Cheung P (2007). Monoubiquitylation of H2A.Z distinguishes its association with euchromatin or facultative heterochromatin. Mol. Cell. Biol..

[41] Otwinowski, Z., Minor, W. & W Jr., C. C. Processing of X-ray diffraction data collected in oscillation mode. (1997). 10.1016/S0076-6879(97)76066-X 27754618

[42] Vagin A, Teplyakov A (2010). Molecular replacement with MOLREP. Acta Crystallogr. D. Biol. Crystallogr..

[43] Adams PD (2010). PHENIX: a comprehensive Python-based system for macromolecular structure solution. Acta Crystallogr. D. Biol. Crystallogr..

[44] Emsley P, Cowtan K (2004). Coot: model-building tools for molecular graphics. Acta Crystallogr. D. Biol. Crystallogr..

[45] Zhang Y (2008). Model-based analysis of ChIP-Seq (MACS). Genome Biol..

[46] Kim D (2013). TopHat2: accurate alignment of transcriptomes in the presence of insertions, deletions and gene fusions. Genome Biol..

[47] Anders S, Pyl PT, Huber W (2015). HTSeq--a Python framework to work with high-throughput sequencing data. Bioinformatics.

[48] McCarthy DJ, Chen Y, Smyth GK (2012). Differential expression analysis of multifactor RNA-Seq experiments with respect to biological variation. Nucleic Acids Res..

[49] Nishino K (2011). DNA methylation dynamics in human induced pluripotent stem cells over time. PLoS Genet..

