# Supplementary material for: Gas41 links histone acetylation to H2A.Z deposition and maintenance of embryonic stem cell identity
Source: Cell Discov. 2018 Jun 12;4:28. doi: 10.1038/s41421-018-0027-0 (PMC5995911; doi:10.1038/s41421-018-0027-0)
Supplement: Supplementary file 1 — Supplementary Information [file 41421_2018_27_MOESM1_ESM.pdf]

Figure S1

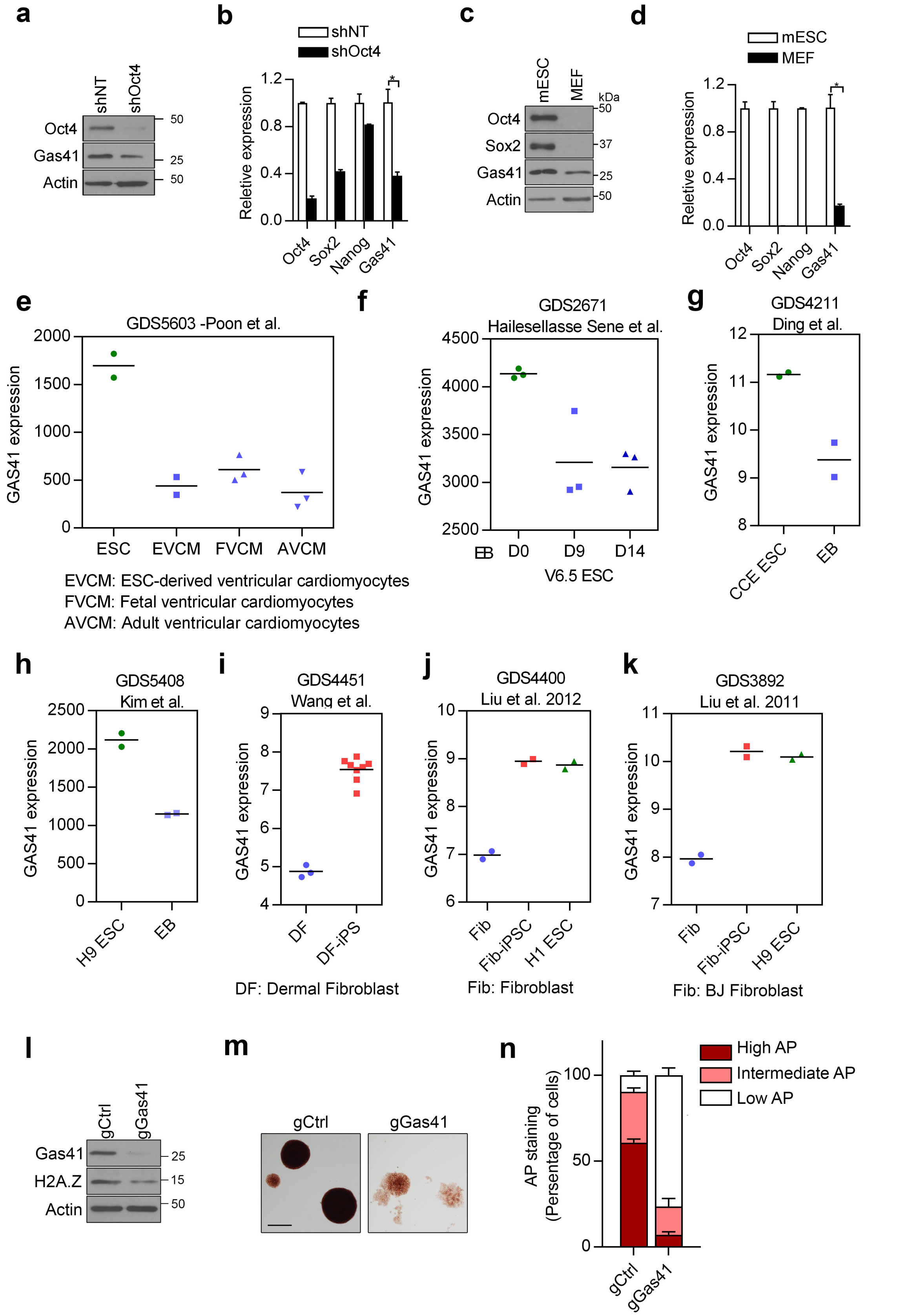
a
shNT
shOct4
Oct4
Gas41
Actin
50
25
50
b
shNT
shOct4
Reletive expression
Oct4
Sox2
Nanog
Gas41
c
mESC
MEF
kDa
Oct4
Sox2
Gas41
Actin
d
mESC
MEF
Reletive expression
e
GDS5603 -Poon et al.
GAS41 expression
ESC
EVCM
FVCM
AVCM
EVCM: ESC-derived ventricular cardiomyocytes
FVCM: Fetal ventricular cardiomyocytes
AVCM: Adult ventricular cardiomyocytes
f
GDS2671
Hailesellasse Sene et al.
EB
D0
D9
D14
V6.5 ESC
g
GDS4211
Ding et al.
CCE ESC
EB
h
GDS5408
Kim et al.
H9 ESC
EB
i
GDS4451
Wang et al.
DF
DF-iPS
DF: Dermal Fibroblast
j
GDS4400
Liu et al. 2012
Fib
Fib-iPSC
H1 ESC
Fib: Fibroblast
k
GDS3892
Liu et al. 2011
Fib
Fib-iPSC
H9 ESC
Fib: BJ Fibroblast
l
gCtrl
gGas41
Gas41
H2A.Z
Actin
m
gCtrl
gGas41
n
AP staining
(Persentage of cells)
High AP
Intermediate AP
Low AP
gCtrl
gGas41

Supplementary Figure S1 ESCs and iPSCs exhibit higher *Gas41* levels than differentiated cells.

(a) Western blot analysis of Gas41 and Oct4 expression in control (shNT) and Oct4 KD mESCs. β-Actin serves as a loading control.

(b) RT–PCR analysis of *Gas41, Oct4, Sox2* and *Nanog* expression levels in control (shNT) and Oct4 KD mESCs. Error bars represent the SEM of n=2. *P<0.05 compared to shNT, two-tailed unpaired Student's t-test.

(c) Western blot analysis of Gas41, Oct4 and Sox2 expression in J1 mESCs and MEFs. β-Actin serves as a loading control.

(d) RT–PCR analysis of *Gas41, Oct4, Sox2* and *Nanog* expression levels in mESCs and MEFs. Error bars represent the SEM of n=2. *P< 0.05 compared to mESCs, two-tailed unpaired Student's t-test.

(**e-k**) Dotplots depicting data extracted from the Gene Expression Omnibus (GEO) Database.

(e) Gas41 expression in HES2 human ESCs, embryonic ventricular cardiomyocytes (EVCM), Fetal ventricular cardiomyocytes (FVCM) and adult ventricular cardiomyocytes (AVCM). Data were extracted from GDS5603 [50].

(f) Time course of Gas41 expression during EB differentiation of V6.5 mESCs. Data were extracted from GDS2671 [51].

(g) Gas41 expression in CCE ESCs and their differentiated EB (Day 10). Data were extracted from GDS4211 [52].

(h) Gas41 expression in H9 human ESCs (hESCs) and their differentiated EB. Data were extracted from GDS5408 [53].

(i) Gas41 expression in dermal fibroblasts (DF) and their induced pluripotent stem (iPS) cells. Data were extracted from GD4451 [54].

(j) Gas41 expression in normal fibroblasts, their iPS cells and H1 hESCs. Data were extracted from GD4400 [55].

(k) Gas41 expression in BJ fibroblasts, their iPS cells and H9 hESCs. Data were extracted from GDS3892 [56].

(l) Western blot analysis Gas41 and H2A.Z expression in control (gCtrl) and Gas41 KO (gGas41) mESCs.

(m)AP staining of control (gCtrl) and Gas41 KO (gGas41) mESCs. Bar, 200 μm.

(n) Stacked bar plot showing quantification of AP staining from (c). Error bars represent the SEM from 10 (gCtrl) or 9 (gGas41) randomly selected fields. Statistical analyses are available in Supplementary table S7.

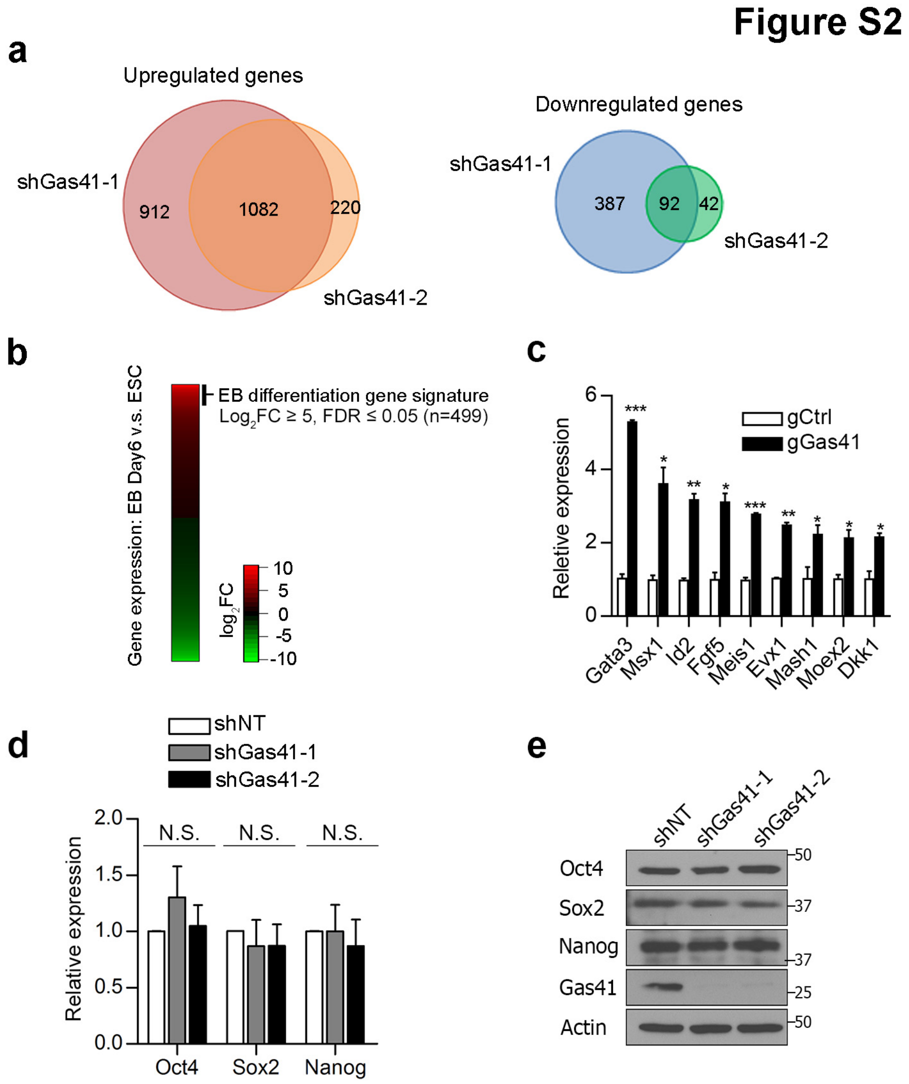

Supplementary Figure S2 Deletion of Gas41 leads to upregulation of developmental genes.
(a) Venn diagrams showing overlaps of upregulated genes (log2FC ⩾ 1 and FDR ⩽ 0.05, left panel) or downregulated genes (log2FC ⩽ -1 and FDR ⩽ 0.05, right panel) in two Gas41 KD mESCs (shGas41-1 and shGas41-2) compared with the control cells (shNT).
(b) Heatmap presentation of expression changes of all genes in differentiated embryoid body (EB, day 6) versus undifferentiated mESCs. The top 499 highly upregulated genes (log2FC ⩾ 5) in EB are defined as the "EB differentiation signature genes".
(c) RT-PCR analysis of expression of selected differentiation marker genes in control (gCtrl) and Gas41 KO (gGas41) mESCs. Error bars represent the SEM of duplicates. *P<0.05, **P<0.01, ***P<0.001 (compared with gCtrl, two-tailed unpaired Student's t-test).
(d) qPCR analysis of mRNA levels of *Oct4, Sox2 and Nanog* in control (shNT) or Gas41 KD (shGas41) mESCs. Error bars represent SEM from n=4 biological independent experiments. N.S., not significant as determined by a Two-tailed unpaired Student's t-test.
(e) Western blot analysis protein levels of Oct4, Sox and Nanog in mESCs KD with indicated Gas41 shRNA.

Figure S3

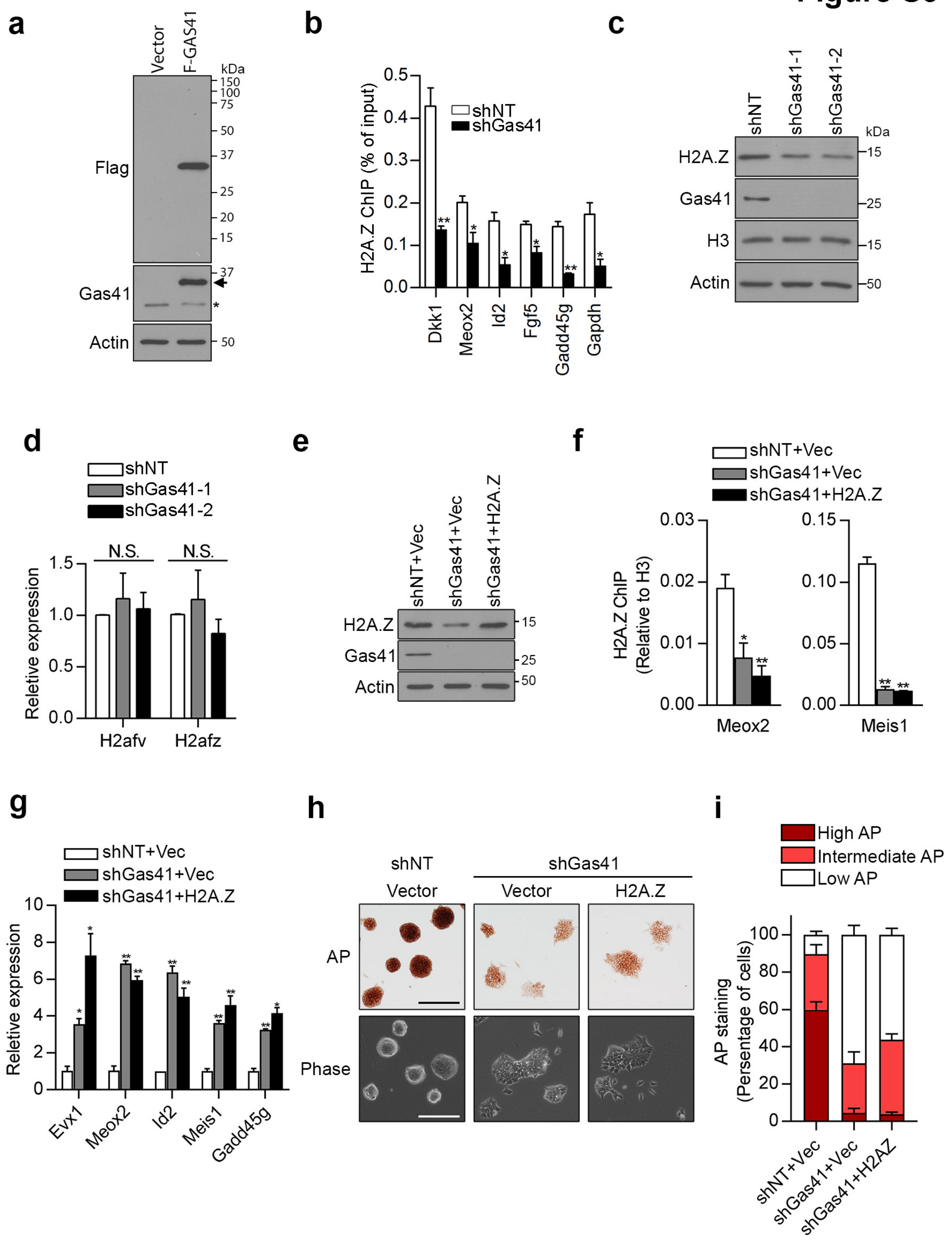

Supplementary Figure S3 Gas41 KD reduces H2A.Z occupancy globally.

(a) Western blot analysis of the Flag-GAS41 stable cells and control cells with the Flag and Gas41 antibodies. Actin is shown as a loading control. Arrow denotes the Flag-tagged GAS41; asterisk denotes endogenous Gas41.

(b) ChIP-qPCR analysis of H2A.Z occupancy in control and Gas41 KD mESCs. Error bars represent the SEM from n=3. *P<0.05, **P<0.01 (compared to shNT, Two-tailed unpaired Student's t-test).

(c) Western blot analysis of H2A.Z levels in control and Gas41 KD mESCs. Histone H3 and β-actin serve as loading controls.

(d) RT-PCR analysis of *H2afv* and *H2afz*, which encode histone H2A.Z, in control and Gas41 KD mESCs. Error bars represent the SEM of n=3 biological independent experiments. N.S., not significant (Two-tailed unpaired Student's t-test).

(e) Western blot analysis of H2A.Z and Gas41 protein levels in shGAS41-1 expressing mESCs rescued with exogenous H2AFZ.

(f) ChIP-qPCR analysis of H2A.Z promoter occupancy of the indicated GAS41-regulated genes in cells in (e). Error bars indicate SEM of n=2 (Meis1) or n=3 (Meox2). *P< 0.05; **P < 0.01 (Compared to shNT, two-tailed unpaired Student's test).

(g) qRT-PCR analysis of indicated Gas41-regulated genes expression in cells in (e). Error bars indicate SEM of n=2. *P<0.05, **P<0.01 (Compared to shNT, Two-tailed unpaired Student's test).

(h) AP staining (upper panel) and phase contrast imaging (lower panel) of cells as in (e). Bar, 200 μM.

(i) Stacked bar plot showing quantification of AP staining from (g). Error bars represent the SEM from 8 (shNT+Vec and shGas41+H2A.Z) or 7 (shGas41+Vec) randomly selected fields. Statistical analyses are available in Supplementary table S7.

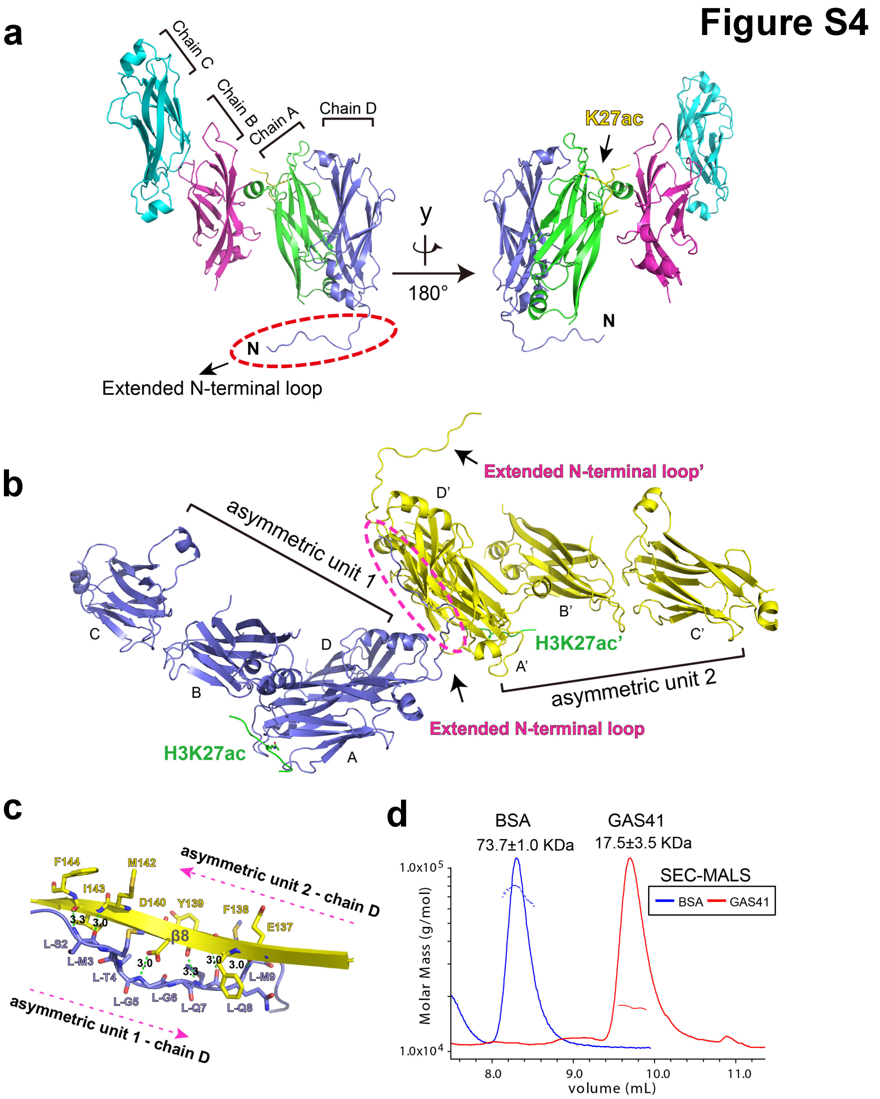

Supplementary Figure S4 Crystal packing and SEC-MALS analysis of Gas41 YEATS domain.

(a) The four-fold asymmetric crystal packing tetramer of Gas41 YEATS in two different viewing angles. The four molecules in one asymmetric unit are shown as ribbon with different colors. Note that only one Gas41 YEATS molecule (Chain D) has an extended N-terminal loop, and only one Gas41 YEATS molecule (Chain A) has the H3 peptide insertion.

(b) Crystal packing mode of two adjacent Gas41 YEATS asymmetry units. The extended N-terminal loop of chain D in asymmetric unit 1 (shown as a purple ribbon) was antiparallel with the β8 strand of chain D' in asymmetric unit 2 (shown as a yellow ribbon). This 'β strand-loop' motif was adopted to mediate crystal packing. The pink dashed circle denotes the interaction surface and with the details depicted in Figure S4c.

(c) The interaction details of the parallel 'β strand-loop' formation between two Gas41 YEATS molecules of adjacent asymmetric units.

(d) Size-exclusion chromatography and multiangle light scattering (SEC-MALS) analysis for the Gas41 YEATS domain in solution. Bovine serum albumin (BSA) is shown as a molecular weight standard in SEC-MALS.

Figure S5

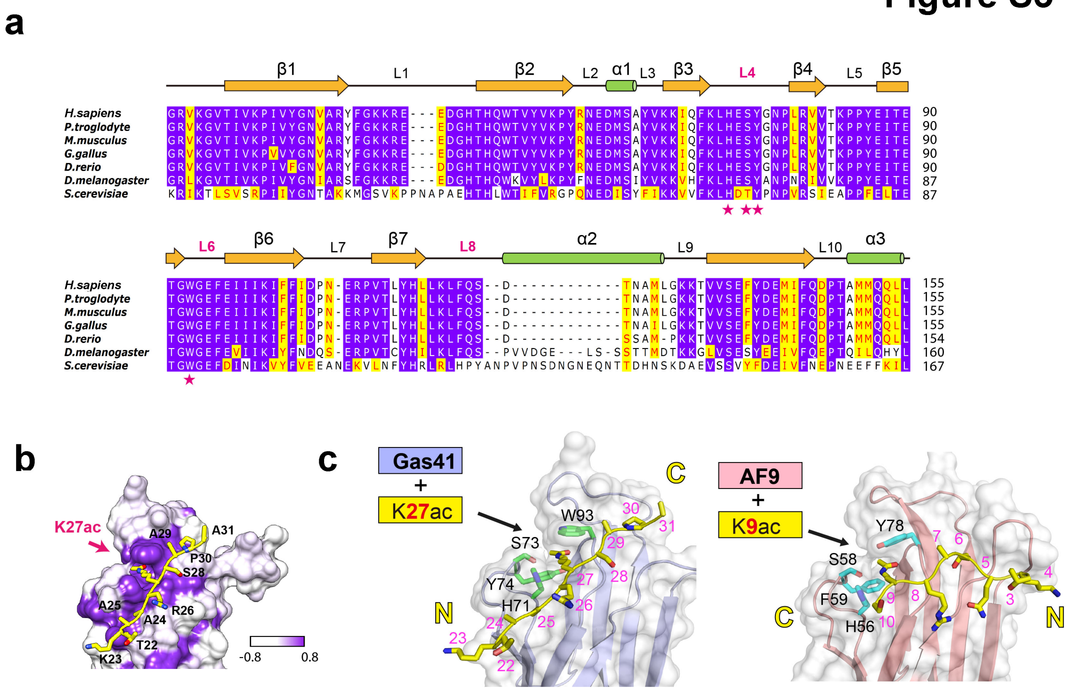

Supplementary Figure S5 The Gas41 YEATS domain recognizes H3K14ac and H3K27ac.
(a) Sequence alignment of the YEATS domain of various Gas41 orthologues. Secondary structure elements of human Gas41 are indicated above the alignment. Conserved residues are shaded in purple; residues involved in binding pocket formation are indicated with red stars.
(b) Conservation mapping around the H3-binding surface among Gas41 orthologues listed in (a). White and purple colors indicate low (-0.8) and high (0.8) sequence conservation, respectively. The H3K27ac peptide is shown as a stick model (yellow).
(c) The orientation of the H3 peptide bound to the YEATS domain. Left panel, the YEATS domain of Gas41 bound to the H3K27ac peptide; right panel, the YEATS domain of AF9 bound to the H3K9ac peptide (PDB ID: 4TMP). The Gas41 YEATS and AF9 YEATS surface views are shown (Gas41, purple ribbon with key residues highlighted as a green stick model; AF9, salmon ribbon with key residues highlighted as a cyan stick model), and the H3 peptides are depicted as a yellow stick model.

Figure S6

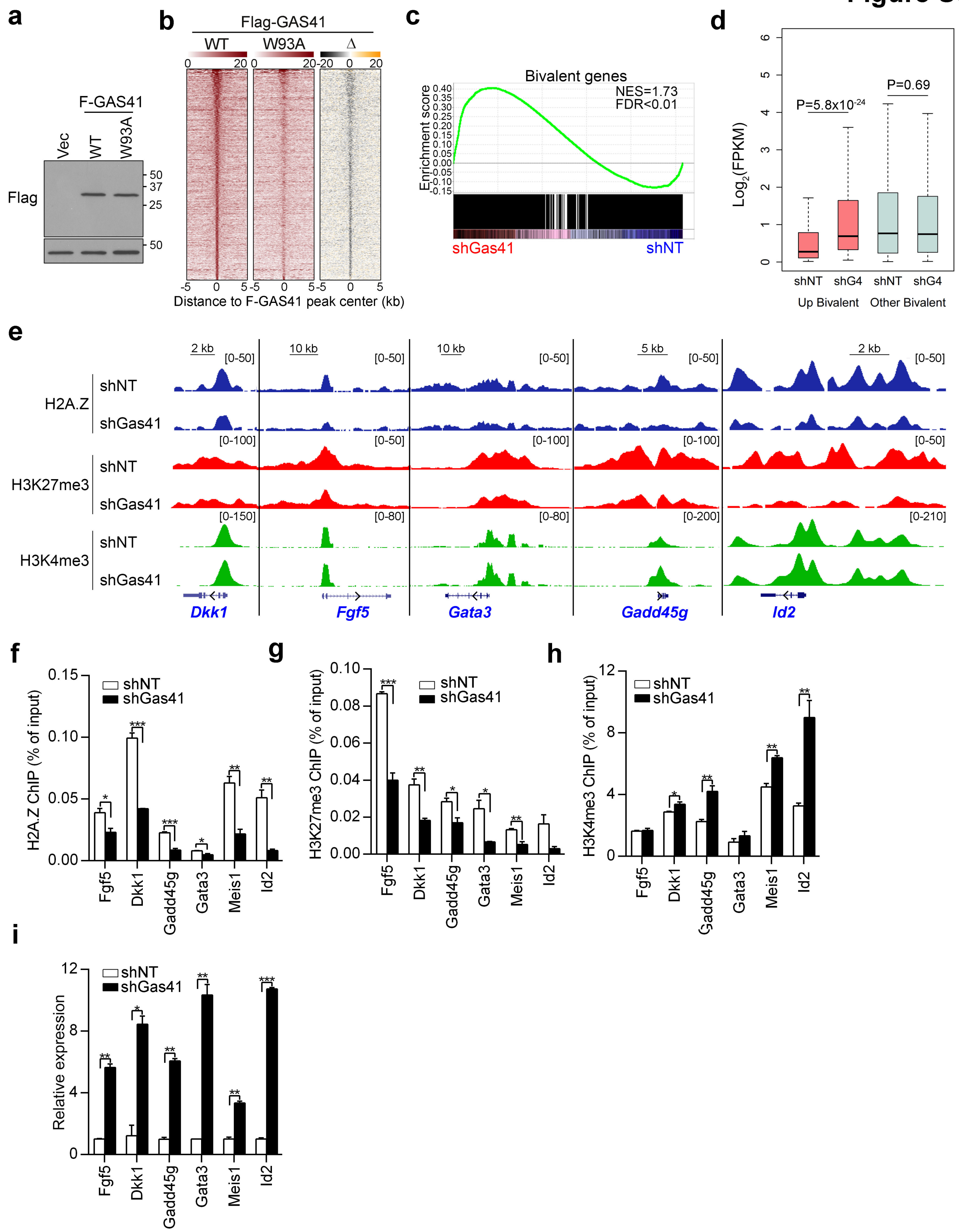

Supplementary Figure S6 Gas41 KD perturbs the balance of bivalent domains.

(a) Western blot analysis of Flag-GAS41 protein levels in mESCs stably expressing WT GAS41 or W93A mutant. β-actin was used as a loading control

(b) Heatmap profiles of WT Flag-GAS41 and W93A mutant occupancies at TSS (±5 kb), and the difference between them (Δ, W93A-WT) in mESCs. Gray indicates a reduction in W93A compared to the WT Flag-GAS41.
(c) GSEA plot of bivalent genes in shGas41 vs the control (shNT) mESCs.
(d) Box plots showing the expression levels of the Up Bivalent and the Other Bivalent genes in control (shNT) and Gas41 KD (shG4) mESCs. The center line represents the median and box limits indicate the 25th and 75th percentiles.
(e) Genome-browser view of the H2A.Z (blue), H3K27me3 (red) and H3K4me3 (green) ChIP-seq peaks on the indicated Up Bivalent genes.
(f-h) ChIP-qPCR analysis of H2A.Z (f), H3K27me3 (g) and H3K4me3 (h) occupancy at promoters of indicated Up bivalent genes in control (shNT) and Gas41 KD (shGas41) mESCs. Error bars represent the SEM of n=3. *P<0.05, **P<0.01, ***P<0.001 (Two-tailed unpaired Student's t-test).
(i) qPCR analysis of mRNA levels of the indicated Up Bivalent genes in control (shNT) and shGas41-1 treated mESCs. Error bars represent the SEM of n=2. *P<0.05, **P<0.01, ***P<0.001 (Two-tailed unpaired Student's t-test).

Figure S7

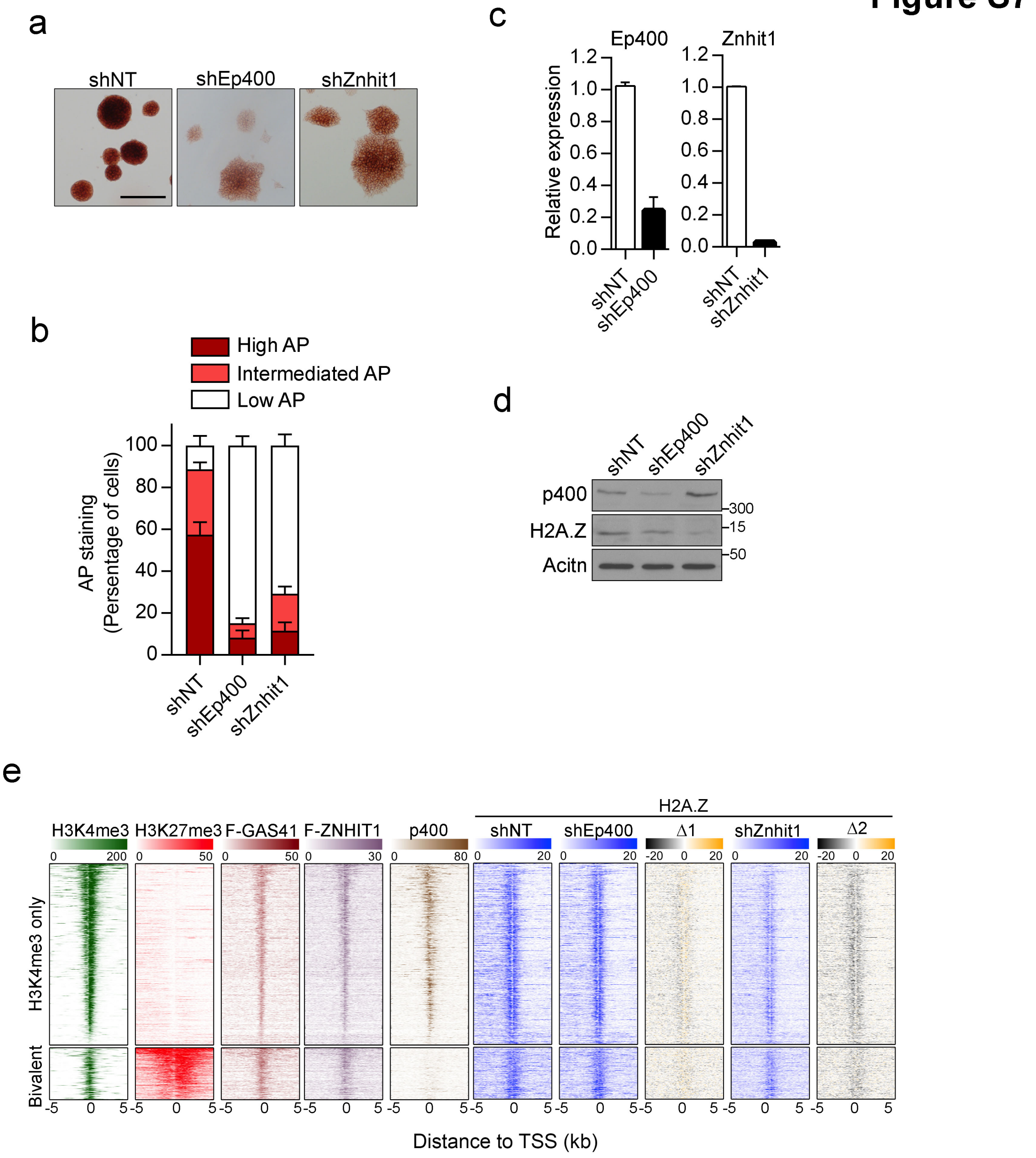

Supplementary Figure S7 H2A.Z occupancy at bivalent promoters is predominantly modulated by SRCAP complex

(a) AP staining of control (shNT), Ep400 (shEp400) and Znhit1 (shZnhit1) KD mESCs. Bar, 200 μM.

(b) Stacked bar plot showing quantification of AP staining from (c). Error bars represent the SEM from 5 (shNT), 6 (shEp400) or 7 (shZnhit1) randomly selected fields. Statistical analyses are available in Supplementary table 7.

(c) qPCR analysis of the KD efficiency of shEp400 (left panel) and shZnhit1 (right panel). Error bars represent the SEM from n=3 biological independent experiments.

(d) Western blot analysis of p400 and H2A.Z expression in control (shNT), Ep400 (shEp400) and Znhit1 (shZnhit1) KD mESCs. β-actin serve as loading controls.

(e) Heatmap profiles of H3K4me3, H3K27me3, Flag-GAS41, Flag-ZNHIT1, and p400 (GSM1581300) occupancies at active promoters (upper panel) or bivalent promoters (lower panel) in mESCs. The H2A.Z occupancy in control (shNT), p400 KD (shEp400), Znhit1 KD (shZnhit1) mESCs, and the difference between them (Δ1, shEp400-shNT; Δ2, shZnhit1-shNT) are also shown. Gray indicates a reduction and yellow indicates an increase in the p400 or Znhit1 KD cells compared to the control (shNT) mESCs.

Supplementary Table S4. Summary of thermodynamic parameters from ITC.

GAS41 (15-159)	Peptides	ΔH (kcal/mol)	ΔS (cal/mol/deg)	Kd (μM)	N
Wild Type	$H3_{1-34}K27ac$	-9.8	-11.1	9.3	1.1
E97A	$H3_{1-34}K27ac$	-8.6	-7.8	13.9	1.0
E95A	$H3_{1-34}K27ac$	-15.3	-32.6	65.4	0.8
S73A	$H3_{1-34}K27ac$	-8.3	-11.1	133	1.0
F96A	$H3_{1-34}K27ac$	-9.9	-18.7	371	1.0
Y74A	$H3_{1-34}K27ac$	-11.4	-24.8	641	1.0
H71A	$H3_{1-34}K27ac$	-17.8	-47.8	877	1.0
G94A	$H3_{1-34}K27ac$	N.D.			
W93A	$H3_{1-34}K27ac$	N.D.			

$H3_{1-34}K27ac$: ARTKQTARKSTGGKAPRKQLATKAARK(ac)SAPATGG
N.D. not detected

SUPPLEMENTARY REFERENCES

50. Poon, E. *et al.* Transcriptome-guided functional analyses reveal novel biological properties and regulatory hierarchy of human embryonic stem cell-derived ventricular cardiomyocytes crucial for maturation. *PLoS One* **8**, e77784 (2013).
51. Hailesellasse Sene, K. *et al.* Gene function in early mouse embryonic stem cell differentiation. *BMC genomics* **8**, 85 (2007).
52. Ding, X., Lin, Q., Ensenat-Waser, R., Rose-John, S. & Zenke, M. Polycomb group protein Bmi1 promotes hematopoietic cell development from embryonic stem cells. *Stem cells and development* **21**, 121-132 (2012).
53. Kim, J.J. *et al.* Discovery of consensus gene signature and intermodular connectivity defining self-renewal of human embryonic stem cells. *Stem cells* **32**, 1468-1479 (2014).
54. Wang, X.M. *et al.* The gene expression profiles of induced pluripotent stem cells from individuals with childhood cerebral adrenoleukodystrophy are consistent with proposed mechanisms of pathogenesis. *Stem cell research & therapy* **3**, 39 (2012).
55. Liu, G.H. *et al.* Progressive degeneration of human neural stem cells caused by pathogenic LRRK2. *Nature* **491**, 603-607 (2012).
56. Liu, G.H. *et al.* Recapitulation of premature ageing with iPSCs from Hutchinson-Gilford progeria syndrome. *Nature* **472**, 221-225 (2011).
